# CpG Islands Recruit a Histone H3 Lysine 36 Demethylase

**DOI:** 10.1016/j.molcel.2010.04.009

**Published:** 2010-04-23

**Authors:** Neil P. Blackledge, Jin C. Zhou, Michael Y. Tolstorukov, Anca M. Farcas, Peter J. Park, Robert J. Klose

**Affiliations:** 1 Department of Biochemistry, University of Oxford, Oxford OX1 3QU, UK; 2 Center for Biomedical Informatics, Harvard Medical School, Boston, MA 02115, USA

**Keywords:** DNA, PROTEINS

## Abstract

In higher eukaryotes, up to 70% of genes have high levels of nonmethylated cytosine/guanine base pairs (CpGs) surrounding promoters and gene regulatory units. These features, called CpG islands, were identified over 20 years ago, but there remains little mechanistic evidence to suggest how these enigmatic elements contribute to promoter function, except that they are refractory to epigenetic silencing by DNA methylation. Here we show that CpG islands directly recruit the H3K36-specific lysine demethylase enzyme KDM2A. Nucleation of KDM2A at these elements results in removal of H3K36 methylation, creating CpG island chromatin that is uniquely depleted of this modification. KDM2A utilizes a zinc finger CxxC (ZF-CxxC) domain that preferentially recognizes nonmethylated CpG DNA, and binding is blocked when the CpG DNA is methylated, thus constraining KDM2A to nonmethylated CpG islands. These data expose a straightforward mechanism through which KDM2A delineates a unique architecture that differentiates CpG island chromatin from bulk chromatin.

## Highlights

► A ZF-CxxC domain in KDM2A specifically binds nonmethylated CpG dinucleotides ► KDM2A is targeted to nonmethylated CpG islands genome-wide ► KDM2A actively removes histone H3 lysine 36 dimethylation from CpG islands ► This unique chromatin architecture distinguishes CpG islands from bulk chromatin

## Introduction

In lower eukaryotes, RNA polymerase II-transcribed promoters are typically compact and contain transcription factor binding sites closely linked to defined polymerase engagement sites (Fuda et al., 2009; Juven-Gershon et al., 2008). In many higher eukaryotes, including humans, a more prevalent class of promoter has evolved that is contained within CpG islands (Bird et al., 1985; Takai and Jones, 2002).

CpG islands were originally biochemically identified using methylation-sensitive restriction enzymes that specifically release nonmethylated regions of the genome from bulk genomic DNA (Bird et al., 1985; Cooper et al., 1983). First called HpaII tiny fragment islands (HTF islands) because of the restriction enzyme used to isolate them (Bird, 1986), these were later renamed CpG islands based on the observation that they corresponded to contiguous nonmethylated regions of the genome that usually had higher than average levels of CpG dinucleotides and GC content (Bird et al., 1985; Bird, 1986; Cooper et al., 1983). These attributes of CpG islands are directly related to their nonmethylated state, as they escape the DNA methylation induced C-to-T transition mutations that accumulate over evolutionary time as a result of 5 methyl-cytosine deamination and imperfect repair (Bird, 1980; Salser, 1977; Tykocinski and Max, 1984). Closer analysis of experimentally identified CpG islands revealed that these elements often overlapped with annotated transcription start sites of genes, suggesting they may contribute to promoter function (Bird et al., 1985; Gardiner-Garden and Frommer, 1987; Tykocinski and Max, 1984). Based on the elevated frequency of CpG and GC content of experimentally isolated CpG islands, computational algorithms have exploited these properties to predict CpG islands genome-wide (Gardiner-Garden and Frommer, 1987; Takai and Jones, 2002). From these predictions it is apparent that gene regulatory elements that are contained within CpG islands encode normal core promoter elements with transcription factor binding sites but differ from compact promoters of lower eukaryotes in that they tend to range in size from a few hundred base pairs to several kilobases, utilize dispersed transcriptional start sites, and are generally more permissive to transcriptional initiation than non-CpG island promoters (Core et al., 2008; Illingworth and Bird, 2009; Juven-Gershon et al., 2008; Ramirez-Carrozzi et al., 2009; Seila et al., 2008; Takai and Jones, 2002). Although the majority of human genes are associated with CpG islands, their role in promoter architecture and function remains poorly understood despite over 20 years of active investigation.

A DNA binding domain, called the ZF-CxxC domain, has been isolated that specifically recognizes nonmethylated CpG dinucleotides in vitro (Voo et al., 2000). The possibility that such a domain may bind CpG islands in vivo is intriguing but has not been tested (Glaser et al., 2006, 2009; Voo et al., 2000). Interestingly, the ZF-CxxC domain is found in proteins involved in regulating chromatin modifications including components of the DNA methylation system (Jorgensen et al., 2004; Pradhan et al., 2008), the H3K4 methyltransferase system (Birke et al., 2002; Lee and Skalnik, 2005), and recently identified H3K36 demethylases (Tsukada et al., 2006). Therefore one potential role for CpG islands could be as a nucleation site for chromatin-modifying proteins that help define a chromatin environment that differentiates these regulatory regions from bulk genomic chromatin.

Based on this possibility, here we investigate the recently identified ZF-CxxC domain containing H3K36me2 demethylase enzyme KDM2A and demonstrate that it binds to nonmethylated CpG islands leading to a depletion of H3K36me2 over these elements. The intrinsic DNA binding specificity of KDM2A provides a simple yet elegant mechanism for recognizing CpG island DNA independently of either transcription factors or the transcriptional state of the associated gene. This permits KDM2A to directly impact chromatin architecture and differentiate CpG island chromatin from bulk chromatin.

## Results

### The KDM2A ZF-CxxC Domain Binds Nonmethylated CpG DNA

**Figure 1:**
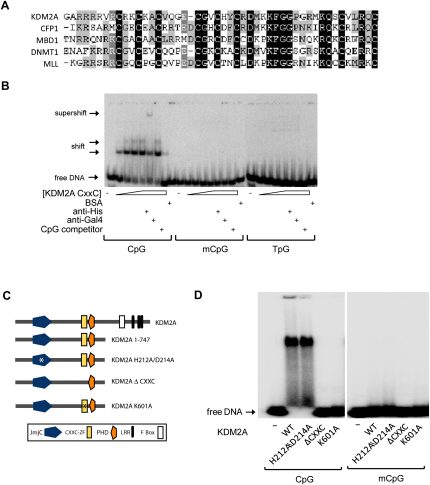
KDM2A Is a Nonmethylated CpG Binding Protein (A) Multiple sequence alignment of the ZF-CxxC domain from KDM2A, CFP1, MBD1, DNMT1, and MLL. (B) A recombinant KDM2A ZF-CxxC domain binds nonmethylated CpG DNA in a protein concentration-dependent manner (left panel), and this association is abrogated by CpG methylation (middle panel) or mutation of the CpGs (right panel) as assessed by EMSA analysis. Binding to nonmethylated CpG is specific, as the interaction is competed with unlabeled competitor DNA and an antibody against the recombinant ZF-CxxC domain supershifts the complex. (C) A schematic illustrating recombinant KDM2A 1–747 and mutant versions of KDM2A, indicating the domain architecture of the protein including the Jumonji C (JmjC), zinc finger CxxC (ZF-CxxC), plant homeodomain-like (PHD), leucine-rich repeat (LRR), and F box (F box) domains. (D) KDM2A binds nonmethylated CpG dinucleotides (left panel, WT), and methylation blocks this binding (right panel, WT) as assessed by EMSA. Mutation of the catalytic JmjC domain (H212A/D214A) does not affect DNA binding, but deletion of the ZF-CxxC domain (ΔCxxC) or a point mutation in the predicted DNA binding face of the ZF-CxxC domain (K601A) abrogates DNA binding.

To understand if CpG islands are a nucleation site for ZF-CxxC domain proteins, we focused on the KDM2 H3K36 demethylase enzymes (Tsukada et al., 2006). These members of the ZF-CxxC domain protein family are particularly interesting, as there is relatively little understanding of how recently identified histone demethylase enzymes are targeted to chromatin substrates (Klose and Zhang, 2007). In mammals there are two KDM2 enzymes. We focused on KDM2A, as KDM2B has a nucleolar localization signal and is concentrated in this specialized compartment (Frescas et al., 2007). Sequence homology analysis revealed that the KDM2A ZF-CxxC domain is conserved with other functional ZF-CxxC domain-containing proteins (Figure 1A), but its capacity to interact with nonmethylated CpG DNA has not been tested. A recombinant KDM2A protein encompassing the ZF-CxxC domain specifically recognized a DNA probe containing two nonmethylated CpGs in an electrophoretic mobility shift assay (EMSA), and binding was abrogated when the CpGs were mutated or methylated (Figure 1B). Importantly, binding to nonmethylated CpG DNA was also detected using a larger version of the protein that included the catalytic Jumonji C (JmjC) domain on longer DNA probes with CpG content similar to that found in CpG islands (Figures 1C and D). The DNA binding specificity of KDM2A was ZF-CxxC domain dependent, as a mutant version of KDM2A lacking the ZF-CxxC domain (ΔCXXC) or with a point mutation in the proposed DNA binding face (K601A) (Allen et al., 2006; Cierpicki et al., 2010) was unable to interact with DNA (Figure 1D). Together these observations demonstrate that KDM2A is a nonmethylated CpG binding protein.

To quantify the interaction of KDM2A with nonmethylated CpG containing DNA, a probe containing one CpG dinucleotide was immobilized on a solid support, and surface plasmon resonance was utilized to determine the dissociation constant (see Figure S1 available online). The K_D_ for KDM2A on this sequence was 0.64 μM, which is similar to the previously measured affinities for a related ZF-CxxC domain interacting with CpG DNA (Allen et al., 2006; Birke et al., 2002; Cierpicki et al., 2010). Because KDM2A will encounter CpG island sequences in vivo that have contiguous regions of DNA containing multiple nonmethylated CpG dinucleotides, a second probe based on the one CpG-containing sequence was engineered by substitution mutations to contain six evenly spaced CpG sites. Interestingly, the K_D_ for KDM2A binding to this probe was 0.56 μM and matched very closely the affinity for the probe containing only one CpG dinucleotide. These observations suggest KDM2A specifically interacts with individual nonmethylated CpG dinucleotides and does not rely on multiple CpGs for binding. This observation is in agreement with a recently published structural study indicating that the ZF-CxxC domain interrogates only one CpG dinucleotide when interacting with DNA (Cierpicki et al., 2010).

### KDM2A Is Recruited via a ZF-CxxC Domain to Nonmethylated DNA In Vivo

**Figure 2:**
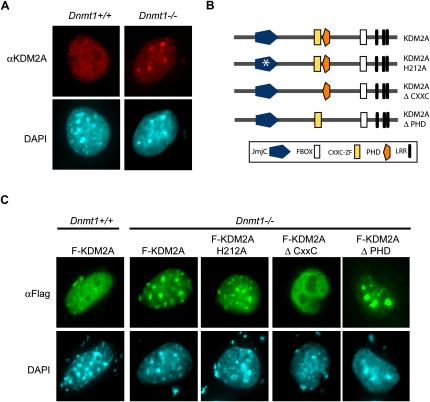
KDM2A Is Targeted to Nonmethylated DNA In Vivo (A) Indirect immunofluoresence with anti-KDM2A antibodies (red) and DAPI staining of DNA (blue). Endogenous KDM2A staining is found throughout the nucleoplasm in *Dnmt*+/+ MEFs (upper left panel). DAPI bright-staining foci mark regions of densely methylated pericentromeric heterochromatin (lower left panel). In *Dnmt1*^−/−^ MEFs almost devoid of CpG methylation, KDM2A localizes to the hypomethylated pericentromeric DAPI bright-staining repeat regions (compare upper and lower right panels). (B) A schematic indicating the epitope-tagged wild-type and mutant versions of KDM2A. (C) Indirect immunofluorescence analyzing localization of exogenous KDM2A expressed in *Dnmt1*^+/+^ (left panel) and *Dnmt1*^−/−^ MEFs (right panels). Deletion of the ZF-CxxC domain (ΔCxxC), but not mutation of the catalytic domain (H212A) or deletion of the PHD domain (ΔPHD), abrogates localization of KDM2A to hypomethylated pericentromeric regions in *Dnmt1*^−/−^ MEFs.

DNA binding analyses in vitro clearly demonstrated that KDM2A recognizes nonmethylated DNA. For KDM2A to recognize CpG islands in vivo, the ZF-CxxC domain would need to target KDM2A to nonmethylated CpGs in native chromatin. To study the chromatin binding and localization properties of endogenous KDM2A, a KDM2A-specific antibody was generated (Figures S2A–S2C). Using this antibody, KDM2A localization was analyzed in mouse embryonic fibroblasts (MEFs) by indirect immunofluorescence. As observed previously with epitope-tagged KDM2A (Frescas et al., 2008; Tsukada et al., 2006), endogenous KDM2A localized to the nucleus and was broadly distributed throughout the nucleoplasm (Figure 2A and Figure S2D). Normal mouse fibroblasts contain large repetitive tracks of densely methylated DNA at pericentromeric heterochromatin that are therefore refractory to binding of ZF-CxxC domain-containing proteins (Jorgensen et al., 2004). In MEFs deficient for the maintenance DNA methyltransferase 1 (*Dnmt1*), these regions of the genome lose DNA methylation (Figure S2J) and become concentrated foci of nonmethylated DNA (Jorgensen et al., 2004; Lande-Diner et al., 2007). In *Dnmt1*-deficient MEFs, KDM2A signal was concentrated at nonmethylated pericentromeric heterochromatin DAPI bright foci in addition to its normal nucleoplasmic staining, indicating that the protein is recruited to nonmethylated DNA in chromatin (Figure 2A and Figure S2E). Importantly, targeting of KDM2A to nonmethylated DNA relied on the ZF-CxxC domain, as an epitope-tagged version of KDM2A (Figure 2B) with a deletion of this domain remained nucleoplasmic in *Dnmt1*-deficient MEFs (Figure 2C and Figure S2H), whereas mutation of other conserved domains in KDM2A had no effect on localization to pericentromeric regions (Figures 2B and 2C and Figures S2F, S2G, and S2I). These observations demonstrate that the ZF-CxxC domain targets KDM2A to nonmethylated chromatin in vivo.

### KDM2A Binds Nonmethylated CpG Island DNA

**Figure 3:**
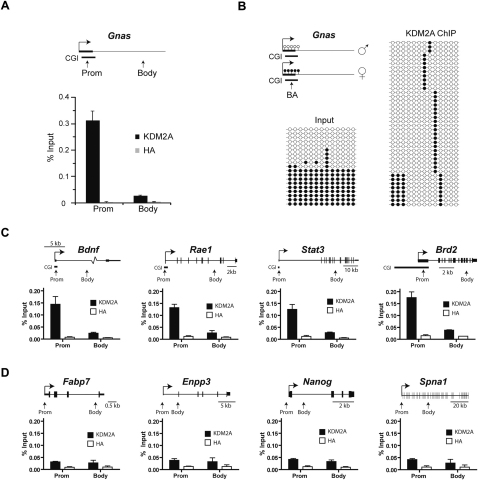
KDM2A Binds to Nonmethylated CpG Islands (A) A schematic of the imprinted *Gnas* gene, which has a CpG island (CGI) promoter (upper panel). KDM2A binds the *Gnas* CpG island as indicated by ChIP with a KDM2A-specific antibody followed by qPCR with promoter and body primer sets (lower panel). ChIP with an HA-specific antibody was used as a negative control. Data are from two biological replicates, and error bars represent SEM. (B) The *Gnas* CpG island is methylated on the maternal allele and nonmethylated on the paternal allele (upper panel) with the bisulfite PCR amplicon indicated (BA). Bisulfite sequencing performed on ChIP input DNA (left panel) and KDM2A ChIP DNA (right panel) indicates that KDM2A preferentially binds the nonmethylated paternal allele. Empty and filled circles represent nonmethylated and methylated CpG dinucleotides, respectively. (C) KDM2A binds to other CpG island promoters. A schematic of each gene is shown, and ChIP data are presented as in (A). (D) KDM2A is not enriched at genes with non-CpG island promoters. ChIP data are presented as in (A).

In the context of a normal cell, the majority of nonmethylated CpG DNA is found in contiguous regions referred to as CpG islands (Bird et al., 1985; Bird, 1986). KDM2A had a clear preference for nonmethylated DNA in vitro and was targeted to nonmethylated pericentromeric DNA in *Dnmt1*-deficient cells. Based on these observations, we sought to examine if KDM2A specifically associated with nonmethylated CpG islands in normal cells. Chromatin immunoprecipitation (ChIP) followed by quantitative PCR (ChIP-qPCR) showed that in mouse embryonic stem cells (ESCs) KDM2A was bound to the CpG island containing *Gnas* promoter but not to the body of the gene (Figure 3A). The *Gnas* gene is subject to an allele-specific epigenetic imprinting process that results in dense methylation of the CpG island on the maternal allele while the paternal allele remains nonmethylated (Figure 3B). Bisulfite DNA sequencing confirmed that this configuration was maintained in our ESC lines (Figure 3B), as approximately 50% of alleles were methylated and 50% were nonmethylated. Strikingly, when the DNA isolated in the KDM2A ChIP was analyzed by bisulfite sequencing, this material was specifically enriched from the nonmethylated allele. This demonstrates that KDM2A associates specifically with the nonmethylated CpG island of the paternal allele in vivo (Figure 3B). Furthermore, when several other CpG island genes were analyzed by ChIP-qPCR, KDM2A was specifically enriched at CpG island promoters, but not at the corresponding gene bodies (Figure 3C and Figure S3). Similar ChIP-qPCR analysis of several genes with non-CpG island promoters showed no KDM2A enrichment (Figure 3D and Figure S3). Together these observations suggest that KDM2A is preferentially targeted to nonmethylated CpG islands.

### KDM2A Associates with CpG Islands Genome-wide

**Figure 4:**
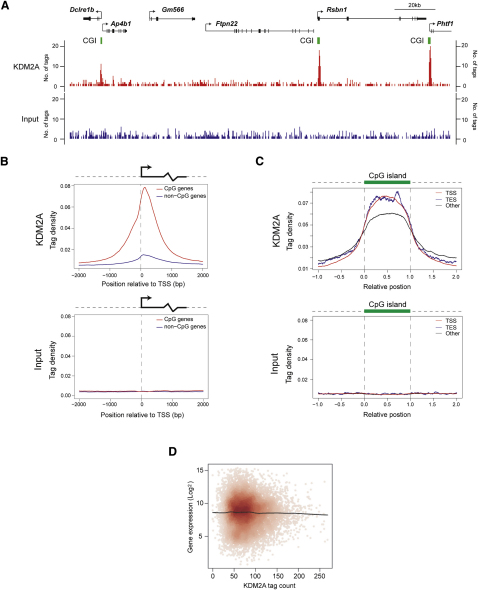
KDM2A Binds to CpG Islands Genome-wide (A) KDM2A ChIP-seq analysis of an approximately 180 kb region of chromosome 3 (chr3:103598131–103782151) in mouse ESCs (top panel, red) with comparative analysis from input material (lower panel, blue). Annotated genes in this region are illustrated above the sequence trace with arrows indicating transcription start sites, vertical black bars corresponding to exons, and CpG islands represented by green bars. (B) Average KDM2A tag density (upper panel) and ChIP input tag density (lower panel) at CpG island-associated transcription start sites (TSS) (red line) compared to non-CpG island TSS (blue line). Density profiles were calculated by averaging tag counts at each position in a 4 kb region around the TSS for all promoters in a set. (C) Average KDM2A tag density (upper panel) and ChIP input tag density (lower panel) across CpG islands at TSS (red line), transcription end sites (TES) (blue line), and other genomic CpG island locations (black line). Tag density is plotted relative to the CpG island coordinates, where the CpG island start and end correspond to positions 0 and 1, respectively. (D) KDM2A binding does not correlate with gene expression levels, as indicted by comparison of gene expression level versus KDM2A tag count in the TSS proximal regions of all CpG island genes. Red dots correspond to individual genes, and a black line representing loess regression is shown to indicate the trend in data distribution. The gene expression levels were estimated based on published microarray data for mouse ESCs (GEO accession number GSE8024) (Mikkelsen et al., 2007).

To assess whether KDM2A association with CpG islands in ESCs is a genome-wide phenomenon, we exploited chromatin immunoprecipitation coupled to massively parallel sequencing (ChIP-seq). After KDM2A-bound DNA was aligned to the mouse genome, a striking overlap was observed between KDM2A binding and algorithm-predicted CpG island elements. This was apparent when we focused on contiguous regions of the genome containing both CpG island and non-CpG island genes with significant KDM2A enrichment only observed at CpG island elements (Figure 4A and Figures S4A–S4D). This remarkable specificity held true when KDM2A tag density was analyzed over all transcription start sites in the genome with specific KDM2A tag density enrichment at bioinformatically defined CpG islands and negligible tag density over non-CpG island promoters (Figure 4B). Importantly, there was no tag density enrichment at either promoter type when the input chromatin sample was analyzed in the same way (Figure 4B). In addition to CpG islands that are associated with gene promoters, some CpG islands are found at transcription end sites or separate from either annotated transcription start or end sites. Similar to the enrichment seen at CpG island promoter regions, KDM2A tag density was enriched over CpG island elements regardless of whether or not they were promoter associated (Figure 4C). Importantly, localization of KDM2A to CpG islands appears to depend on the presence of underlying nonmethylated CpG DNA sequence and not simply as a secondary consequence of engagement of the transcriptional machinery, as KDM2A-specific enrichment was observed at both expressed and nonexpressed CpG island genes (Figures S4E–S4G).

Based on ChIP-seq analysis, KDM2A occupies greater than 90% of CpG islands and over 95% of transcription start site-associated CpG islands as defined by the CpG island prediction algorithm (Table S1). Importantly, these regions encompass the majority of high-magnitude KDM2A binding clusters (Figure S4H, Figure 4A, Figures S4A–S4D). Algorithm-based peak finding also identified a large fraction of low-magnitude KDM2A-enriched regions that occurred outside of bioinformatically annotated CpG islands (Table S1). To investigate whether these binding events occurred at nonmethylated regions of the genome, a series of non-CpG island KDM2A ChIP-seq peaks (both promoter associated and non-promoter associated) were analyzed by bisulfite sequencing (Figures S4L–S4Q). The analyzed promoter-associated non-CpG island KDM2A peaks correspond to contiguous regions of nonmethylated DNA (Figures S4L and S4M), indicating they are likely CpG islands that fall below the criteria for inclusion in the algorithm-defined CpG island set. This suggests that a proportion of bona fide nonmethylated CpG islands has been excluded from the CpG islands prediction set, as has been observed previously (Illingworth et al., 2008). This is also in fitting with the observation that non-CpG island KDM2A peaks tend to exhibit GC content and observed/expected CpG ratios greater than that of bulk genomic DNA (Figures S4J and S4K). Other KDM2A sites tended to be of very low magnitude, and bisulfite sequencing verified that these regions only contain small amounts of mosaically nonmethylated DNA (Figures S4N–S4Q). Therefore, in fitting with the observation that the majority of nonmethylated DNA is found in CpG islands, strong KDM2A binding corresponds to annotated CpG islands (Figure S4H), and KDM2A tag density is more concentrated at strong as opposed to weak CpG islands (Figure S4I).

Together these observations demonstrate that KDM2A recognizes CpG islands through an intrinsic DNA binding capacity as opposed to relying on nucleation through transcription factors, as do other histone demethylases and most chromatin-modifying enzymes. Because KDM2A does not rely on the transcriptional regulatory machinery to interact with DNA sequences, one would envisage that binding and therefore function at CpG island genes was independent of the underlying transcriptional state of the associated gene. This is supported by the fact that KDM2A enrichment was detected at over 95% of transcription start sites that contain CpG islands, but tag density did not correlate with gene expression levels of these CpG island genes in mouse ESCs (Figure 4D). Furthermore, KDM2A binds both expressed and nonexpressed CpG island genes (Figures S4E–S4G). These observations are particularly important as direct binding of KDM2A to CpG island DNA suggests a function for this protein that does not directly result in transcriptional activation or repression per se.

### CpG Islands Are Specifically Depleted of H3K36me2

**Figure 5:**
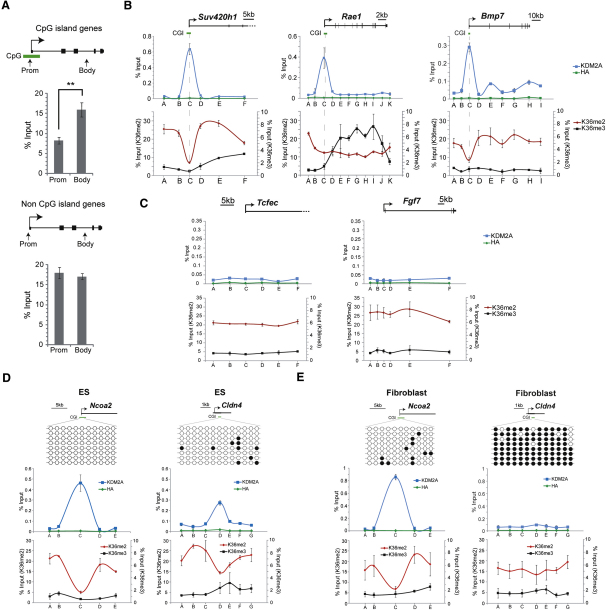
Depletion of H3K36me2 at KDM2A-Bound CpG Islands (A) H3K36me2 is depleted at KDM2A-bound CpG islands as indicated by the average H3K36me2 ChIP enrichments for a series of CpG island (upper panel) and non-CpG island genes (lower panel). For each type of gene, qPCR primer sets specific to promoter and body regions were used. For each primer set, H3K36me2-specific enrichment was normalized to histone H3 occupancy. Each data set represents the average of nine individual genes (see Figure S5). At CpG island genes, K36me2 is significantly depleted at the promoter relative to the body region (^∗∗^p < 0.01), whereas at genes with non-CpG island promoters there is no significant difference in K36me2 between promoter and body regions (p > 0.05). (B and C) (B) Comparison of KDM2A (upper panel, blue line) and H3K36me2/me3 (lower panel, red and black lines) ChIP profiles across tiled CpG island genes and (C) non-CpG island genes, indicating specific enrichment of KDM2A and depletion of H3K36me2 at CpG islands. Individual genes are depicted to scale above each panel. Anti HA antibody (green line) is included as a negative control in the KDM2A ChIP experiments. Enrichment values for H3K36me2 (left-hand y axis) and me3 (right-hand y axis) are normalized to histone H3 occupancy. (D and E) (D) Comparison of DNA methylation by bisulfite sequencing (upper panel), KDM2A occupancy by ChIP (middle panel), and H3K36me2/me3 levels by ChIP (bottom panel) at *Ncoa2* and *Cldn4* in mouse ESCs and (E) mouse fibroblasts. Empty and filled circles (upper panel) represent nonmethylated and methylated CpG dinucleotides, respectively. KDM2A ChIP and H3K36me2/me3 ChIP are represented as described for (B) and (C). DNA methylation of the *Cldn4* CpG island in fibroblasts blocks KDM2A binding and abrogates the depletion of H3K36me2 over the CpG island region that is observed in ESCs, whereas the *Ncoa2* CpG island remains nonmethylated in both cell types, retains KDM2A, and has a depletion of H3K36me2 at the CpG island. All ChIP data represent at least two biological replicates, and error bars indicate SEM.

A potential role for KDM2A at CpG islands could be to mark these regions of the genome by specifically depleting H3K36 methylation. KDM2A is a histone H3 lysine 36 (H3K36) demethylase that preferentially removes the dimethyl (me2), and to a lesser extent the monomethyl (me1), modification state (Tsukada et al., 2006). To explore if H3K36 methylation was depleted at CpG island promoters, ChIP was carried out at a series of CpG island and non-CpG island genes, and H3K36me1, -me2, and -me3 modifications were analyzed. Remarkably, the levels of H3K36me1 and -me2 were significantly depleted in the promoter regions of CpG island genes when compared to the promoters of non-CpG island genes and the body of both classes of genes (Figure 5A and Figures S5A and S5B). Depletion of H3K36me2 was most clearly observed at CpG island promoters, in line with the previously characterized enzymatic preference of KDM2A toward this modification state (Tsukada et al., 2006) and our observations by ChIP-seq that KDM2A binds CpG islands genome-wide. To understand if the H3K36me2 depletion at CpG island promoters corresponded precisely to regions of KDM2A binding, KDM2A and H3K36me2 ChIP profiles were compared over a series of tiled amplicons covering genes with CpG island and non-CpG island promoters (Figures 5B and 5C). Importantly, at CpG island-containing promoters, KDM2A binding peaked over the CpG island region and precisely corresponded to the region of chromatin that showed depletion of H3K36me2 (Figure 5B). In clear contrast, non-CpG island promoters had no KDM2A and lacked obvious H3K36me2 depletion (Figure 5C). Interestingly, over the body of expressed genes, depletion of H3K36me2 was also observed, and this corresponded to the appearance of the H3K36me3 state (Figure 5B). This may represent conversion of H3K36me2 to the -me3 modification state, in agreement with previous observations in ESCs that H3K36me3 occurs exclusively over the body of actively transcribed genes (Mikkelsen et al., 2007) and the recent discovery in fly and mouse that the H3K36me2 and -me3 modification states are placed by different enzymes (Bell et al., 2007; Edmunds et al., 2008). Surprisingly, we observed relatively constant levels of H3K36me2 upstream, over the body, and downstream of genes in all regions analyzed. This suggested that H3K36me2 may be more broadly distributed in chromatin of higher eukaryotes than previously realized and parallels published mass spectrometry data that indicated that up to 40% of mammalian cellular histone H3 was characterized by the H3K36me2 state (Garcia et al., 2008; Peters et al., 2003; Robin et al., 2007). Because KDM2A was targeted to CpG islands based on nonmethylated DNA sequence, it appears to function in a unique and generic manner to remove H3K36me2 from island regions and epigenetically differentiate these regulatory elements from bulk chromatin and non-CpG island promoters.

### Depletion of H3K36me2 Is Lost when KDM2A Binding Is Inhibited by CpG Island Methylation

KDM2A binding to CpG DNA in vitro is blocked by DNA methylation, and it preferentially associates with nonmethylated CpG islands in vivo (Figures 1B, 1D, and 3B). Although a clear attribute of CpG islands is their capacity to remain free of DNA methylation whether the associated gene is active or repressed, as cells differentiate during development a small number of CpG island promoters undergo de novo DNA methylation through a poorly understood mechanism. Because DNA methylation blocks KDM2A binding, this permitted chromatin analysis at a defined gene either in the presence or absence of KDM2A. To this end, we examined H3K36me2 at a control gene with a CpG island that is nonmethylated in both ESCs and differentiated fibroblasts (*Ncoa2*) and a gene that has a CpG island that acquires DNA methylation in fibroblasts (*Cldn4*) (Figures 5D and 5E). In mouse ESCs and fibroblasts, the *Ncoa2* CpG island promoter was nonmethylated, bound KDM2A, and showed specific depletion of H3K36me2 over the CpG island (Figures 5D and 5E). In stark contrast, while the nonmethylated *Cldn4* CpG island also showed KDM2A binding and H3K36me2 depletion in ESCs, acquisition of CpG island DNA methylation in differentiated fibroblasts correlated with the absence of KDM2A binding and the appearance of H3K36me2 at the CpG island (Figure 5E, right panel). Therefore, KDM2A binding correlates with CpG island-specific depletion of H3K36me2. Strikingly, this observation also highlights that cell-type-specific epigenetic changes in CpG island methylation can regulate KDM2A binding and thus impact promoter-specific H3K36 modification.

### Depletion of KDM2A Causes Increases in H3K36me2 at CpG Islands

**Figure 6:**
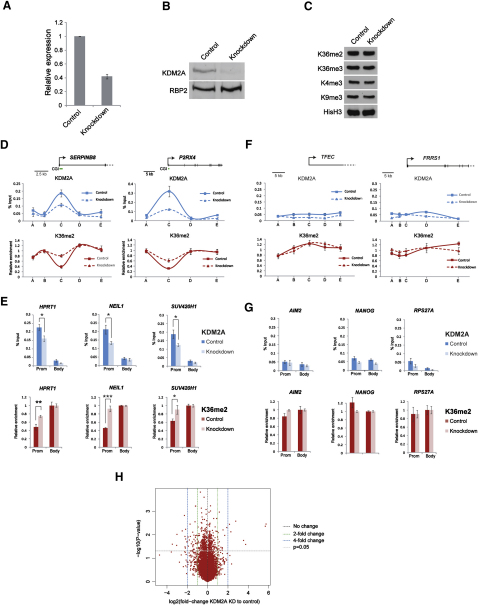
Depletion of KDM2A Causes Increases in H3K36me2 at CpG Islands (A) A KDM2A knockdown cell line displays an approximately 60% decrease in KDM2A mRNA levels compared to the control cell line. (B) Western blot with an antibody against KDM2A shows depletion of KDM2A protein levels in the KDM2A knockdown cell line compared to control cell line. Western blot with an RBP2 antibody demonstrates equal loading. (C) KDM2A knockdown cells exhibit no gross differences in levels of H3K36me2, H3K36me3, H3K4me3, or H3K9me3 relative to control cells. Western blot with a histone H3 antibody demonstrates equal loading. (D) Shown is comparison of KDM2A (upper panel blue lines) and H3K36me2 (lower panel red lines) ChIP profiles across tiled CpG island genes in control (solid lines) and KDM2A knockdown cell line (dashed lines). KDM2A knockdown cells exhibit specific loss of KDM2A enrichment at CpG islands, resulting in a CpG island-specific gain of H3K36me2. Individual genes are depicted to scale above each panel. (E) Comparison of KDM2A (upper panel, blue bars) and H3K36me2 (lower panel, red bars) ChIP at promoter and body regions of CpG island genes in control (dark blue and dark red) and KDM2A knockdown cells (light blue and light red). At CpG island regions, the KDM2A knockdown cell line shows a significant loss of KDM2A-specific enrichment compared to control cells (^∗^p < 0.05). Loss of KDM2A at CpG islands correlates with a significant H3K36me2 increase in knockdown cells compared to control (^∗^p < 0.05, ^∗∗^p < 0.01, ^∗∗∗^p < 0.001). (F and G) Non-CpG island genes were analyzed as in (D) and (E). At genes with non-CpG island promoters, KDM2A knockdown cells show no significant difference in KDM2A or K36me2 enrichment compared to control cells (p > 0.05). All ChIP data represent at least two biological replicates, and error bars indicate SEM. (H) Volcano plot comparison of expression values in the KDM2A knockdown and control cell lines reveals subtle but significant changes in gene expression. Fold change in expression values (KDM2A knockdown relative to control) and the statistical significance of the differences (two sided t test) were computed for each transcript interrogated on the array (red dots). Vertical dashed black line represents zero change in expression, vertical dashed green lines represent a 2-fold change in expression, and vertical dashed blue lines represent a 4-fold change in expression. Transcript data points above the horizontal dashed gray line show a statistically significant change in expression between KDM2A knockdown and control cell lines (p < 0.05).

Despite exhaustive attempts to knock down KDM2A in a number of different ES and primary cell lines using an array of RNAi-mediated approaches, KDM2A has proved to be highly refractory to depletion, suggesting it may contribute to an essential process in these cell types. To try and circumvent this limitation, a stably expressed shRNA-mediated approach was used to deplete KDM2A in human cervical carcinoma cells. Approximately 60% knockdown was achieved at the RNA level as assessed by RT-PCR (Figure 6A), and clear depletion of the protein was observed when assessed by western blot analysis (Figure 6B). To understand if KDM2A depletion affected global levels of H3K36me2, bulk cellular histone was isolated and histone methylation levels analyzed by western blot (Figure 6C). No obvious global changes in H3K36me2 or other histone methylation marks were observed, in fitting with the contention that H3K36me2 is an abundant modification and KDM2A-mediated depletion is restricted to a small fraction of the genome. To understand if KDM2A contributes to the depletion of H3K36me2 at CpG islands, ChIP-qPCR was used to analyze KDM2A and H3K36me2 occupancy in the control and KDM2A knockdown cell lines (Figures 6D–6G). Importantly, in the control line, KDM2A occupancy was restricted to CpG island sequences and H3K36me2 was depleted at CpG islands, as observed in mouse ES and fibroblast cells (Figures 6D and 6E). In the KDM2A knockdown cell line, we observed a reduction in KDM2A ChIP signal specifically at CpG islands genes (Figures 6D and 6E). The reduction of KDM2A at CpG island genes resulted in a significant increase in H3K36me2 levels specifically at the CpG island. Importantly, this reduction in KDM2A occupancy and increase in H3K36me2 in the KDM2A knockdown line was not due to alteration in the underlying DNA methylation profiles due to perturbed KDM2A levels (Figures S6A and S6B). In contrast, we did not observe any significant increase in the levels of H3K36me2 at non-CpG island genes (Figures 6F and 6G). Together these observations demonstrate that depletion of KDM2A results in encroachment of H3K36me2 into CpG islands regions and indicate a causative role for KDM2A demethylase activity in defining depletion of this mark at CpG islands.

When the transcription levels of CpG island genes were analyzed comparing the control and KDM2A knockdown line by RT-PCR, there was no general correlation between the depletion of KDM2A and upregulation or downregulation of steady-state transcript levels (Figure S6C). This is in fitting with the observation in mouse ESCs that KDM2A does not specifically bind to expressed or nonexpressed CpG island genes and argues against KDM2A playing a role as a traditional activator or repressor. We did observe some subtle changes in gene expression between the control and KDM2A KD cell line by RT-PCR (Figure S6C). This prompted an analysis of the genome-wide expression profiles in the control and KDM2A knockdown cell lines by microarray-based gene expression profiling (Figure 6H). Interestingly, this analysis revealed statistically significant changes in gene expression, but the majority of these were subtle and less than 2-fold. KDM2A potentially associates with up to two-thirds of genes through association with CpG islands, but we did not observe any clear correlation between these gene expression changes and CpG island or non-CpG island genes (data not shown), suggesting that any CpG island-specific changes are indiscernible from downstream secondary systemic effects. As elaborated on in the discussion, removal of H3K36me2 by KDM2A as part of the systems that define CpG island chromatin architecture may contribute to the transcriptional competency of CpG island genes. Consistent with this possibility, depletion of KDM2A causes subtle changes in gene expression that appear to be tolerated by these transformed cells. One possible explanation for the fact that KDM2A depletion has been unattainable in primary nontransformed cells could be that these cell types are unable to tolerate the aggregated effect of the gene expression perturbations resulting from KDM2A depletion, even though these are moderate in magnitude. Nevertheless, these observations suggest that KDM2A is not functioning as a potent CpG island-specific transcriptional activator or repressor but instead defines chromatin architecture at CpG islands through enzymatic removal of H3K36me2, a process that may indirectly contribute to normal transcriptional profiles.

## Discussion

**Figure 7:**
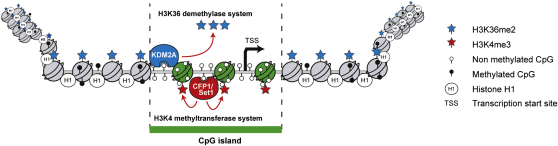
Unique Chromatin Architecture at CpG Islands CpG islands utilize nonmethylated CpG dinucleotides to recruit the ZF-CxxC domain protein KDM2A, depleting these regions of H3K36me2 and creating a chromatin environment that is unique from bulk non-CpG island chromatin. CpG island chromatin is also modified by H3K4me3, and this relies on targeting of the Set1 complex by the ZF-CxxC domain containing CFP1 protein (A. Bird, personal communication). Another facet of CpG island chromatin is depletion of histone H1. Because CpG islands are usually part of regulatory regions or associated with transcription start sites, this unique chromatin architecture may function to differentiate these regulatory regions from bulk chromatin in large complex mammalian genomes.

Although the existence of CpG islands has been known for over two decades, the mechanisms through which they contribute to genome function have remained poorly understood. Here we provide compelling evidence that CpG islands are actively recognized by KDM2A binding to nonmethylated CpG DNA through a ZF-CxxC domain (Figures 1–3). Using ChIP-seq we show that KDM2A binds to 90% CpG island elements genome-wide and that this nucleation event is independent of the transcriptional status of the associated gene (Figure 4). We demonstrate that KDM2A occupancy at CpG islands imposes a unique H3K36me2-depleted chromatin signature that differentiates these regulatory elements from bulk chromatin, and we provide functional data demonstrating that depletion of KDM2A results in H3K36me2 spreading into CpG island elements (Figures 5 and 6). Together these observations provide insight into how CpG islands can impact chromatin architecture at regulatory elements by utilizing their underlying DNA sequence as a nucleation site for a chromatin-modifying enzyme (Figure 7). Therefore, these data suggest a central function of CpG island DNA may be to impose a chromatin architecture that differentiates CpG island chromatin from bulk chromatin, highlighting these important regulatory regions within large and complex mammalian genomes. In support of this contention, CpG island chromatin is also enriched in H3K4me3 regardless of whether the associated gene is expressed (Bernstein et al., 2006; Guenther et al., 2007). Intriguingly, Set1 and Mll H3K4 methyltransferase complexes that trigger this modification also have ZF-CxxC domains (Birke et al., 2002; Lee and Skalnik, 2005), suggesting they may use a similar mechanism as KDM2A to target CpG islands (Figure 7). This possibility is supported by evidence that the ZF-CxxC domain-containing protein, CFP1, which is a component of the Set1 H3K4 methyltransferase complex, is targeted to CpG island elements genome-wide, where it deposits H3K4me3 methylation in a manner that is independent of the transcriptional state of the associated gene (A. Bird, personal communication). These observations regarding CFP1 nucleation and H3K4me3 perfectly mirror our discovery that KDM2A binds CpG islands, resulting in enzymatic depletion of H3K36me2 independently of the transcriptional state of the associated gene. Together these data indicate that ZF-CxxC domain-containing proteins are important mediators of CpG island chromatin architecture and highlight the fact that CpG islands as a DNA-encoded genetic element are functioning to directly impact the epigenetic state of surrounding chromatin.

Interestingly, the processes that impose a unique chromatin environment at CpG islands are presumably even more complex. For example, from biochemical assays it is known that CpG island chromatin is specifically depleted of linker histone H1 (Tazi and Bird, 1990) (Figure 7). Given that CpG island chromatin architecture appears to form irrespective of transcriptional state, an interesting question is what impact this chromatin environment has on the function of associated genes. It is known that H3K36me2 and H1 can have an inhibitory effect on transcription initiation (Carrozza et al., 2005; Cheung et al., 2002; Levine et al., 1993; Li et al., 2009; Strahl et al., 2002; Youdell et al., 2008), and H3K4me3 is generally associated with processes positively contributing to transcription (Kouzarides, 2007). Therefore, one possibility is that unique CpG island chromatin architecture reinforced by several intersecting chromatin-regulating pathways may define regions of the genome that are more permissive to nucleation of the transcriptional machinery, effectively differentiating CpG islands from bulk chromatin and highlighting regulatory regions of the genome. The inherent complexity of the CpG chromatin signature makes this a difficult hypothesis to directly examine experimentally, but this concept is indirectly supported by the observation in genome-wide run-on transcription assays that CpG island promoters sustain nonproductive transcriptional initiation events in both sense and antisense directions even in the absence of activated directional transcription, whereas non-CpG island genes fail to show this property (Core et al., 2008; Seila et al., 2008). An important implication of this hypothesis is that CpG island chromatin architecture would provide an environment that is permissive to transcription but not drive productive directional transcriptional output, a process that requires transcription factor binding and concerted gene activation mechanisms. This type of CpG island-specific transcriptional competence has recently been shown to contribute to the induction kinetics of CpG island-containing genes in activated macrophages (Ramirez-Carrozzi et al., 2009).

In conclusion, we have provided a mechanistic link between CpG island elements and nucleation of a histone demethylase and demonstrated that CpG islands function to define cellular chromatin landscapes at these regulatory elements. A corollary of this observation from a genome evolution standpoint is that ZF-CxxC domain recognition of CpG island elements may also impose an additional selective pressure over evolutionary time to maintain the nonmethylated state of CpG island elements. Based on our understanding of CpG island function presented here, a future challenge will be to understand if chromatin modifications at CpG islands impact the transcriptional machinery and how non-CpG island promoters differ in the absence of these mechanisms.

## Experimental Procedures

### Cell Culture

Cell types used in the study were cultured as described in the Supplemental Experimental Procedures.

### Chromatin Immunoprecipitation

For KDM2A, ChIP cells were fixed for 1 hr in 2 mM EGS, followed by 15 min in 1% formaldehyde. For histone modification, ChIP cells were fixed for 10 min in 1% formaldehyde alone. A detailed ChIP protocol, including information about specific antibodies used for ChIP, is described in the Supplemental Experimental Procedures.

### ChIP Sequencing

ChIP material was sequenced using a Solexa 2G instrument. Sequencing data are available at GEO accession number GSE21202. Sequencing data were analyzed as described in the Supplemental Experimental Procedures.

### Immunofluoresence

Cells were fixed and immunostained with specific antibodies, as described in the Supplemental Experimental Procedures. Photographs were captured with an AxioSkop fluorescent microscope (Zeiss).

### Protein Expression and Purification

DNA constructs used for protein expression are described in the Supplemental Experimental Procedures. Expression and purification of KDM2A ZF-CxxC domain and KDM2A 1–747 constructs were performed as previously described (Klose and Bird, 2004) except for minor modifications described in the Supplemental Experimental Procedures.

### Electrophoretic Mobility Shift Assay and Surface Plasmon Resonance

Detailed protocols for EMSA and surface plasmon resonance experiments, including information about probes, are described in the Supplemental Experimental Procedures.

### Microarray and Data Analysis

RNA labeling and hybridization to 4 × 44 K human gene expression microarrays (Agilent Technologies, Inc., Santa Clara, CA) were carried out by Oxford Gene Technology (Oxford, UK). Hybridizations were performed for two biological replicates for each sample. Microarray data are available at GEO accession number GSE21202. Microarray data were analyzed as described in the Supplemental Experimental Procedures.

## Accession Numbers

ChIP sequencing and microarray expression data are available at GEO accession number GSE21202.

## Supplemental Information

Document S1. Six Figures, One Table, Supplemental Experimental Procedures, and Supplemental References
