# Supplementary material for: CpG Islands Recruit a Histone H3 Lysine 36 Demethylase
Source: Mol Cell. 2010 Apr 23;38(2-2):179–90. doi: 10.1016/j.molcel.2010.04.009 (PMC3098377; doi:10.1016/j.molcel.2010.04.009)
Supplement: Document S1. Six Figures, One Table, Supplemental Experimental Procedures, and Supplemental References [file mmc1.pdf]

Molecular Cell, Volume *38*

Supplemental Information

CpG Islands Recruit a Histone H3 Lysine 36 Demethylase

Neil P. Blackledge, Jin C. Zhou, Michael Y. Tolstorukov, Anca M. Farcas, Peter J. Park, and Robert J. Klose

Supplemental Information Index
Blackledge et al.

Supplemental Figures (pages 2-11)

Figure S1- page 2

Figure S2- page 3-4

Figure S3- page 5

Figure S4- page 6-8

Figure S5- page 9-10

Figure S6- page 11

Supplemental Figure Legends (pages 12-17)

Supplemental Table 1 (page 18)

Supplemental Experimental Procedures (pages 19-25)

Supplemental References (page 26)

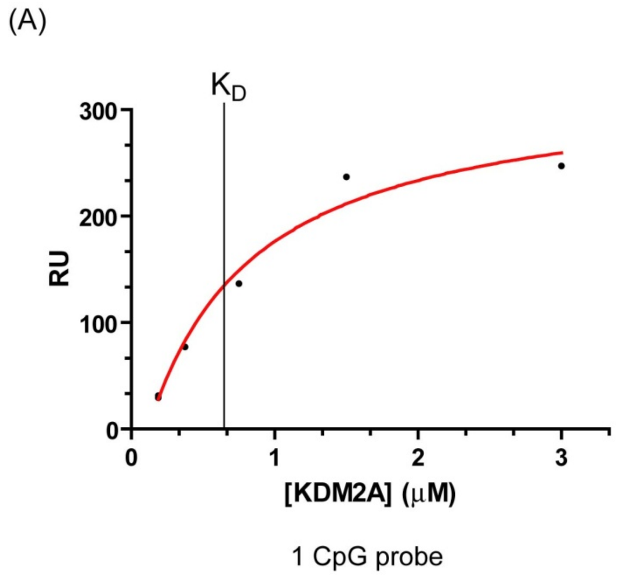

Figure S1

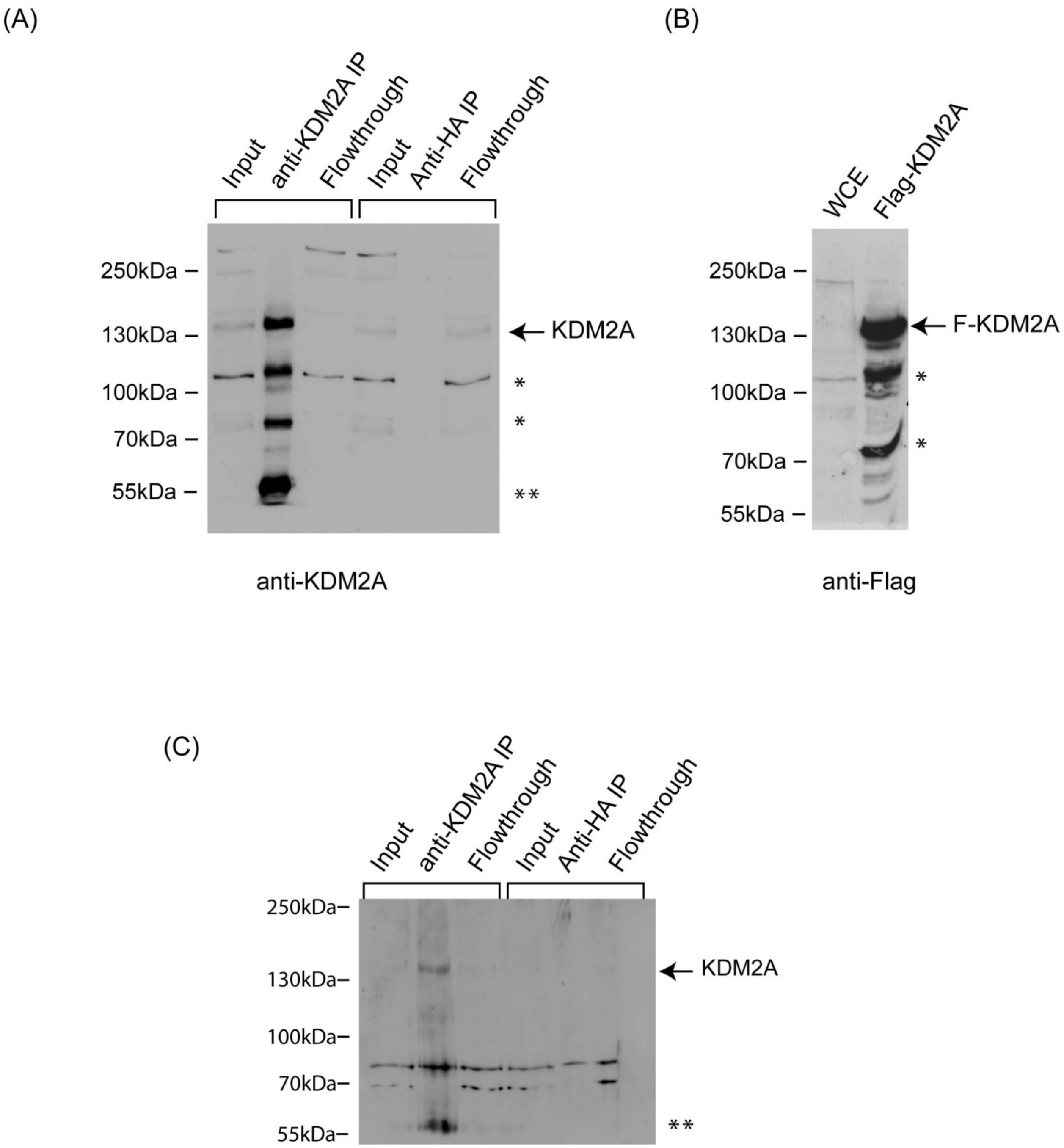
(A)
Input
anti-KDM2A IP
Flowthrough
Input
Anti-HA IP
Flowthrough
250kDa
130kDa
100kDa
70kDa
55kDa
KDM2A
*
*
**
anti-KDM2A
(B)
WCE
Flag-KDM2A
250kDa
130kDa
100kDa
70kDa
55kDa
F-KDM2A
*
*
anti-Flag
(C)
Input
anti-KDM2A IP
Flowthrough
Input
Anti-HA IP
Flowthrough
250kDa
130kDa
100kDa
70kDa
55kDa
KDM2A
**

Figure S2

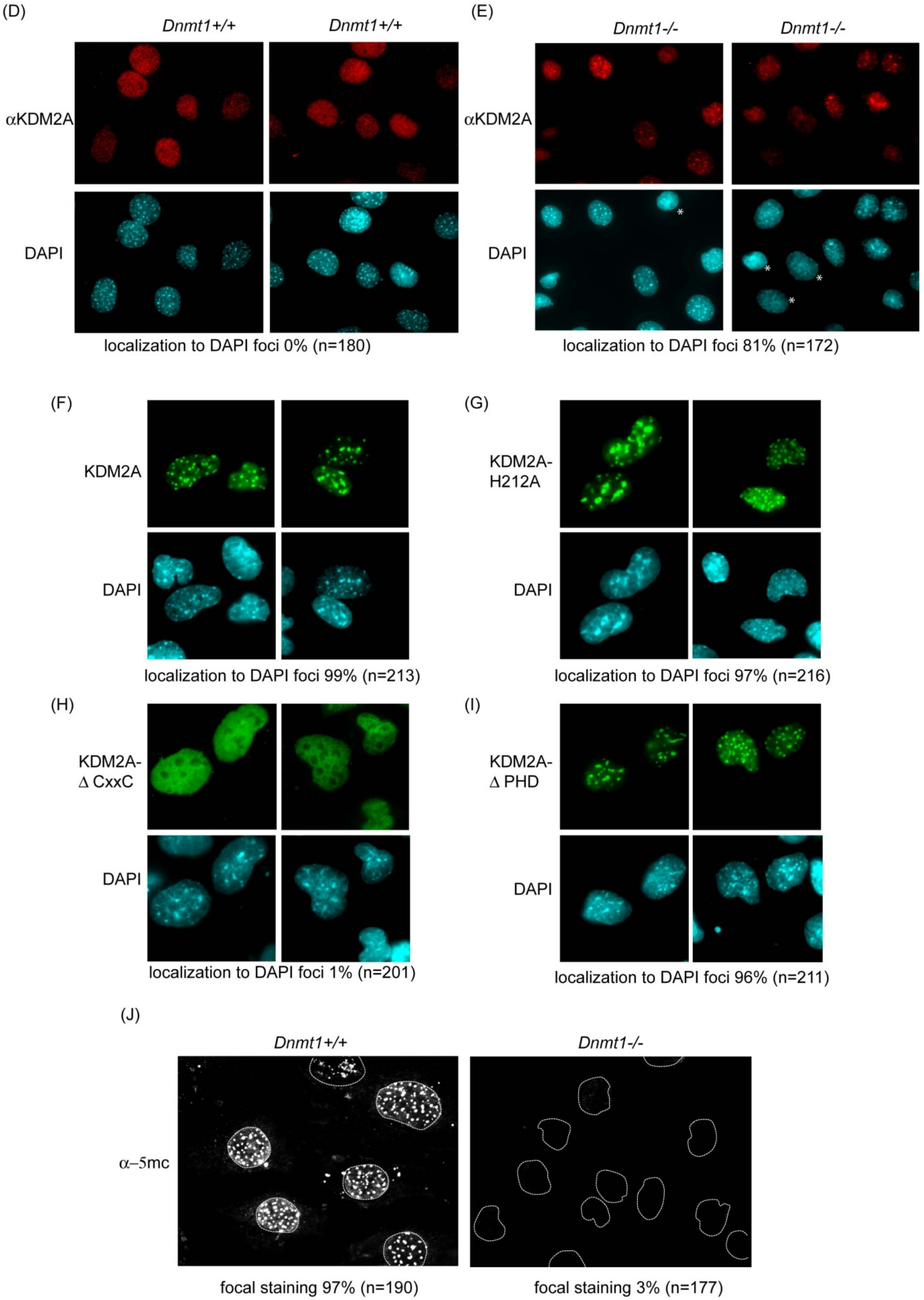

Figure S2 (continued)

CpG island genes

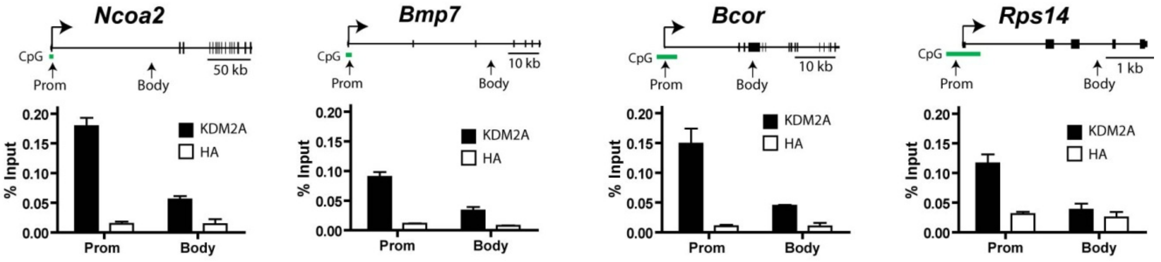

Non CpG island genes

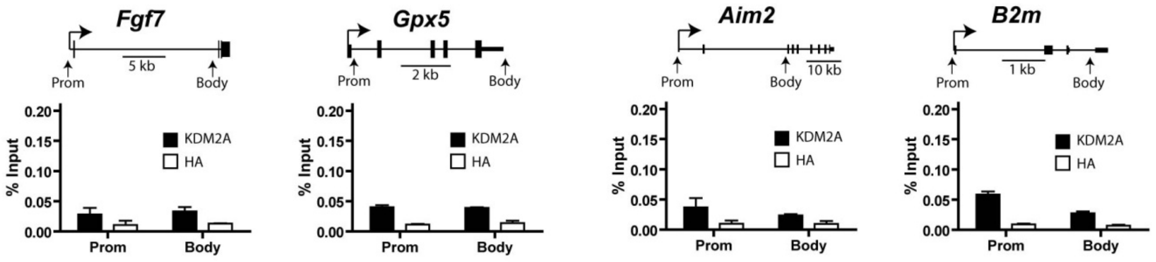

Figure S3

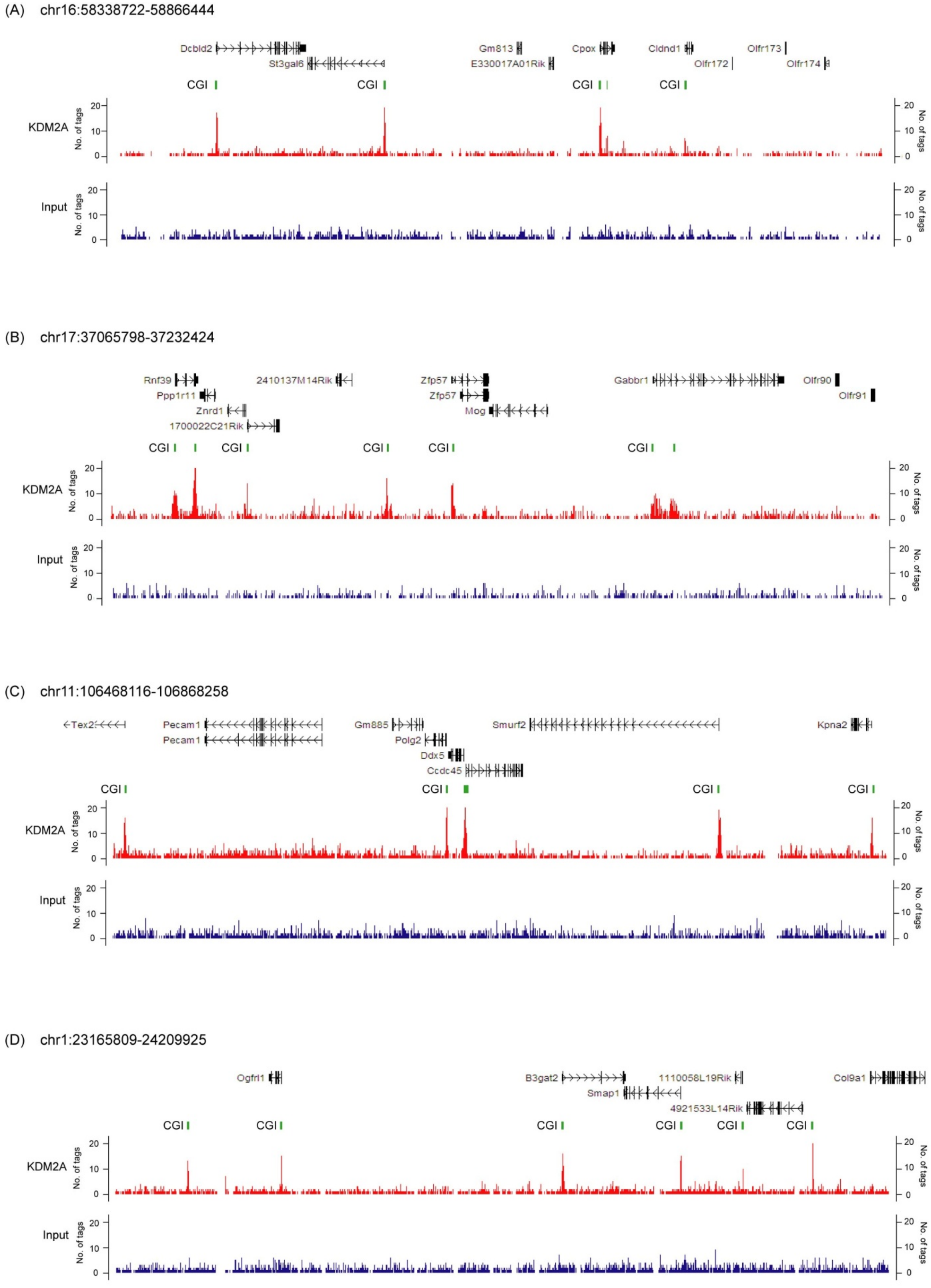
(A) chr16:58338722-58866444
Dcbld2
St3gal6
Gm813
E330017A01Rik
Cpox
Cldnd1
Olfr172
Olfr173
Olfr174
CGI
KDM2A
Input
No. of tags
(B) chr17:37065798-37232424
Rnf39
Ppp1r11
Znrd1
1700022C21Rik
2410137M14Rik
Zfp57
Mog
Gabbr1
Olfr90
Olfr91
CGI
KDM2A
Input
No. of tags
(C) chr11:106468116-106868258
Tex2
Pecam1
Gm885
Polg2
Ddx5
Ccdc45
Smurf2
Kpna2
CGI
KDM2A
Input
No. of tags
(D) chr1:23165809-24209925
Ogfrl1
B3gat2
Smap1
1110058L19Rik
4921533L14Rik
Col9a1
CGI
KDM2A
Input
No. of tags

Figure S4

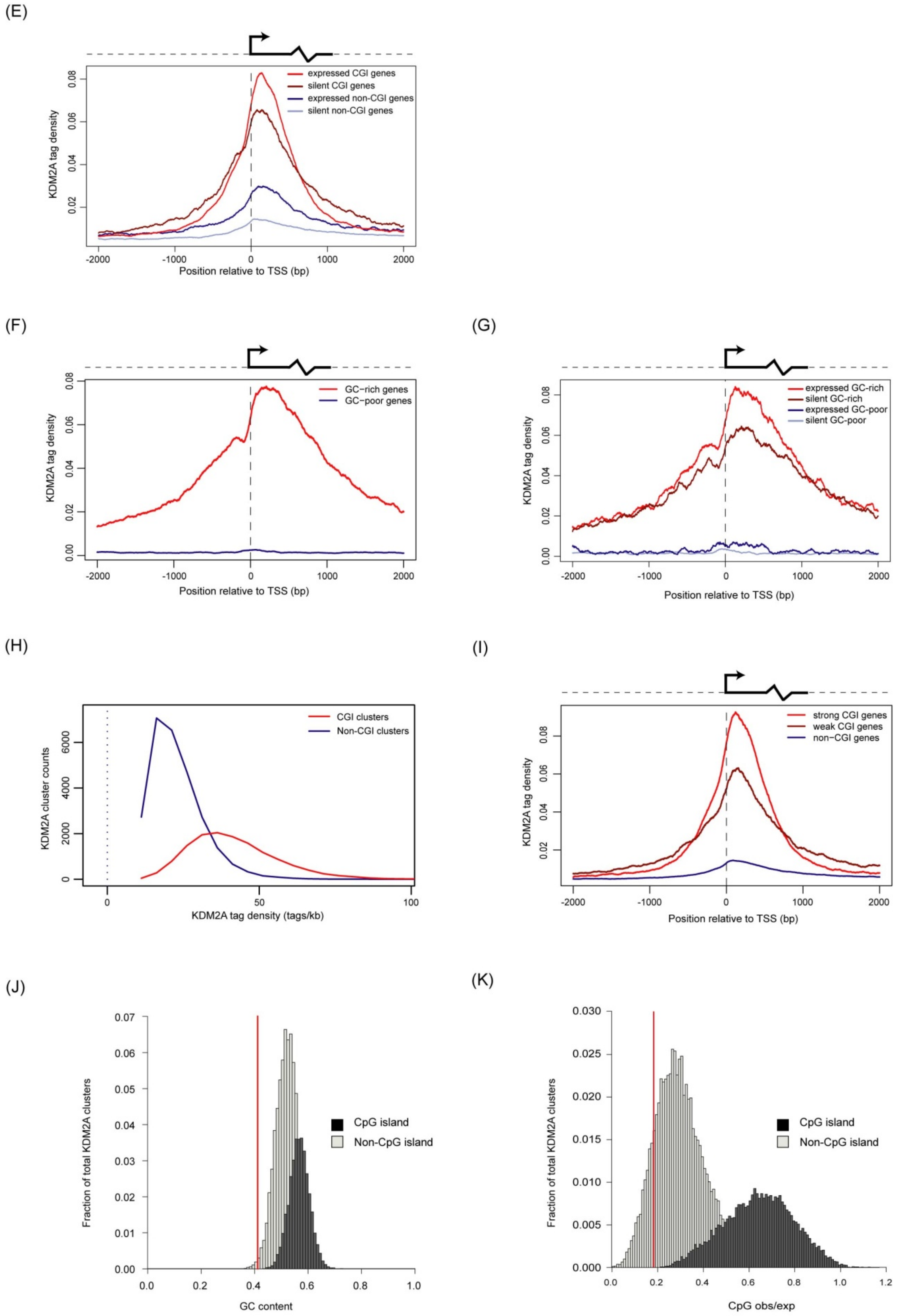

Figure S4 (continued)

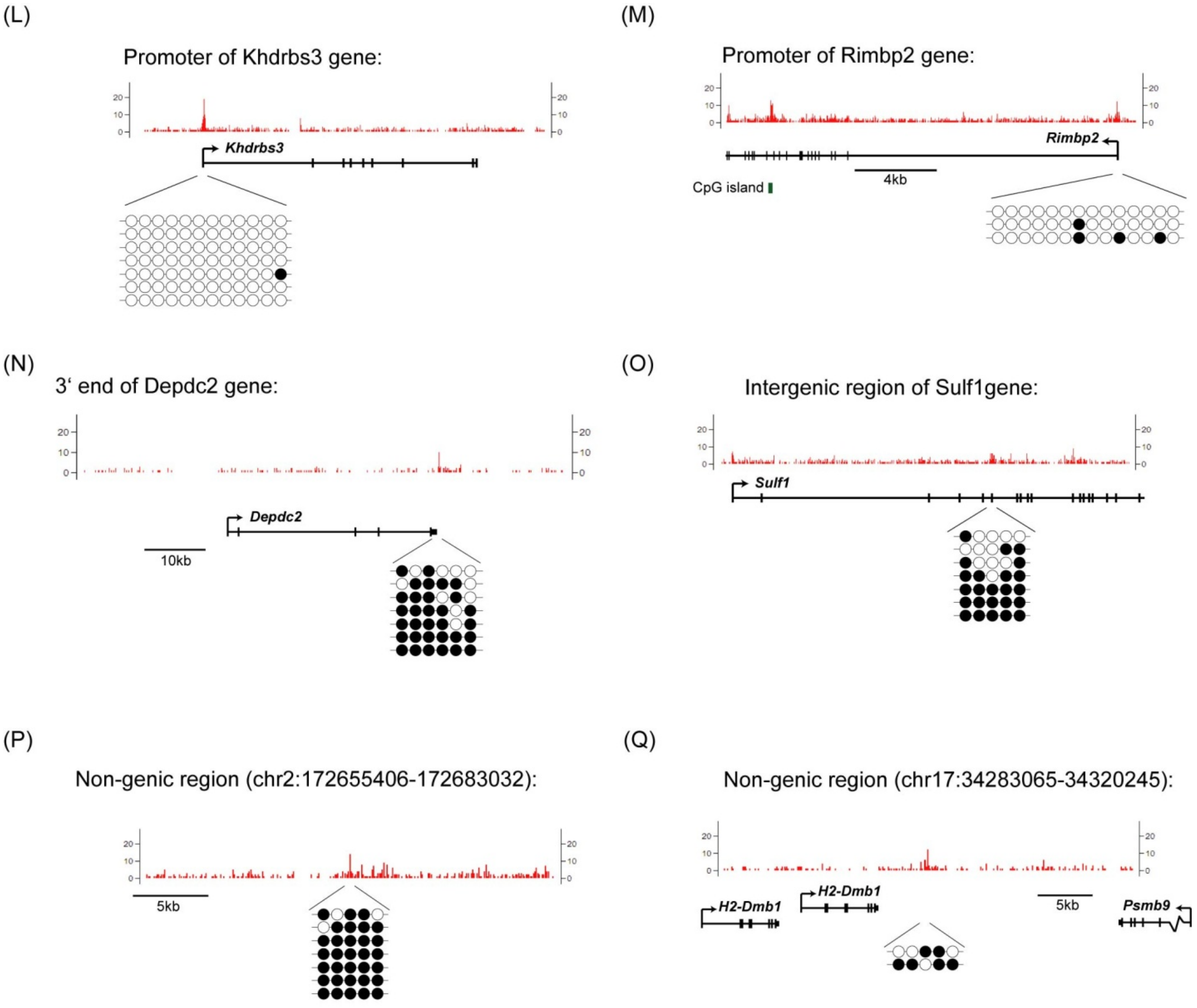

Figure S4 (continued)

(A)

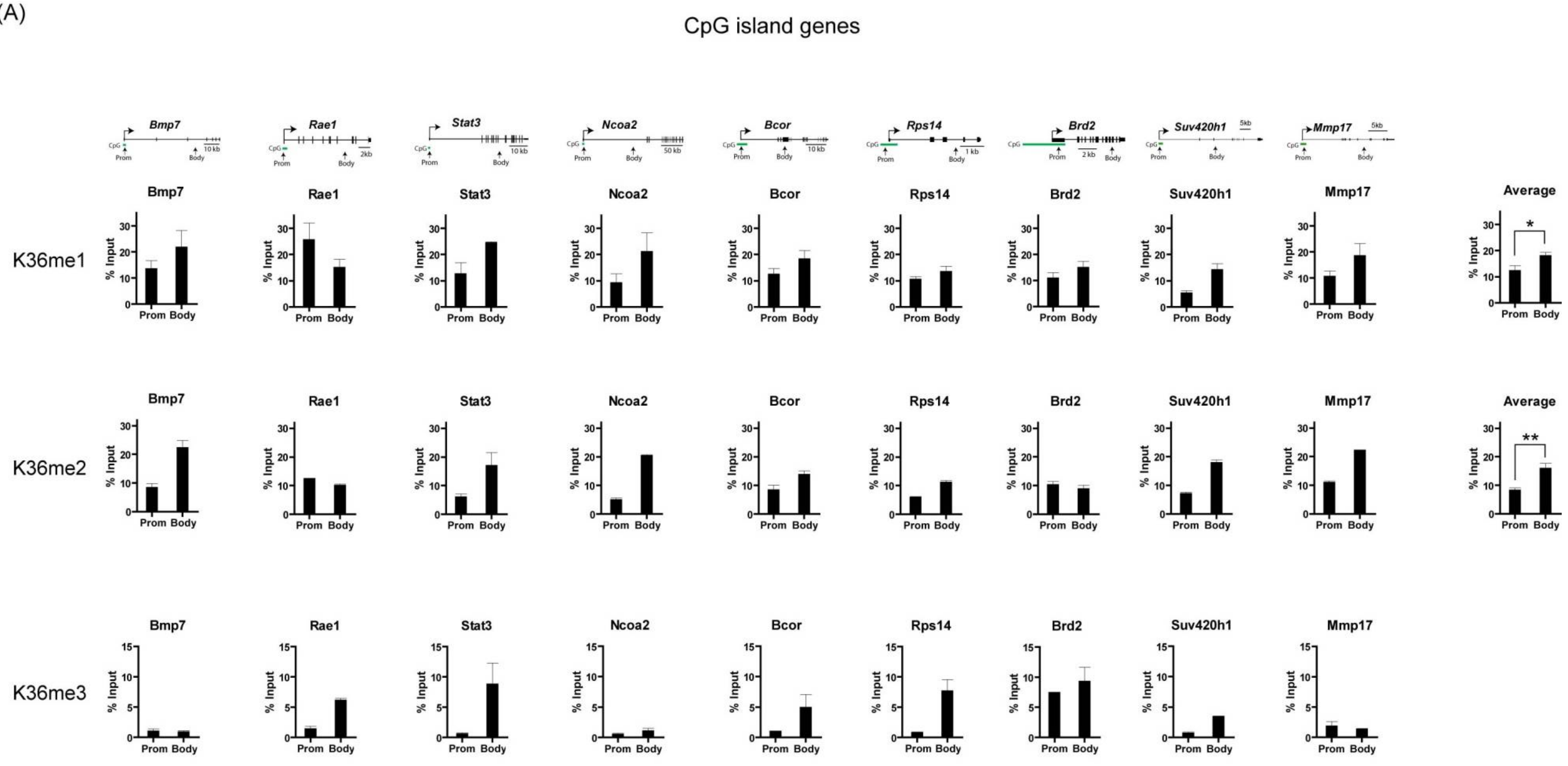
CpG island genes
K36me1
K36me2
K36me3
Bmp7
Rae1
Stat3
Ncoa2
Bcor
Rps14
Brd2
Suv420h1
Mmp17
Average
% Input
Prom Body
CpG
Prom
Body
10 kb
2kb
50 kb
1 kb
2 kb
5kb
*
**

Figure S5

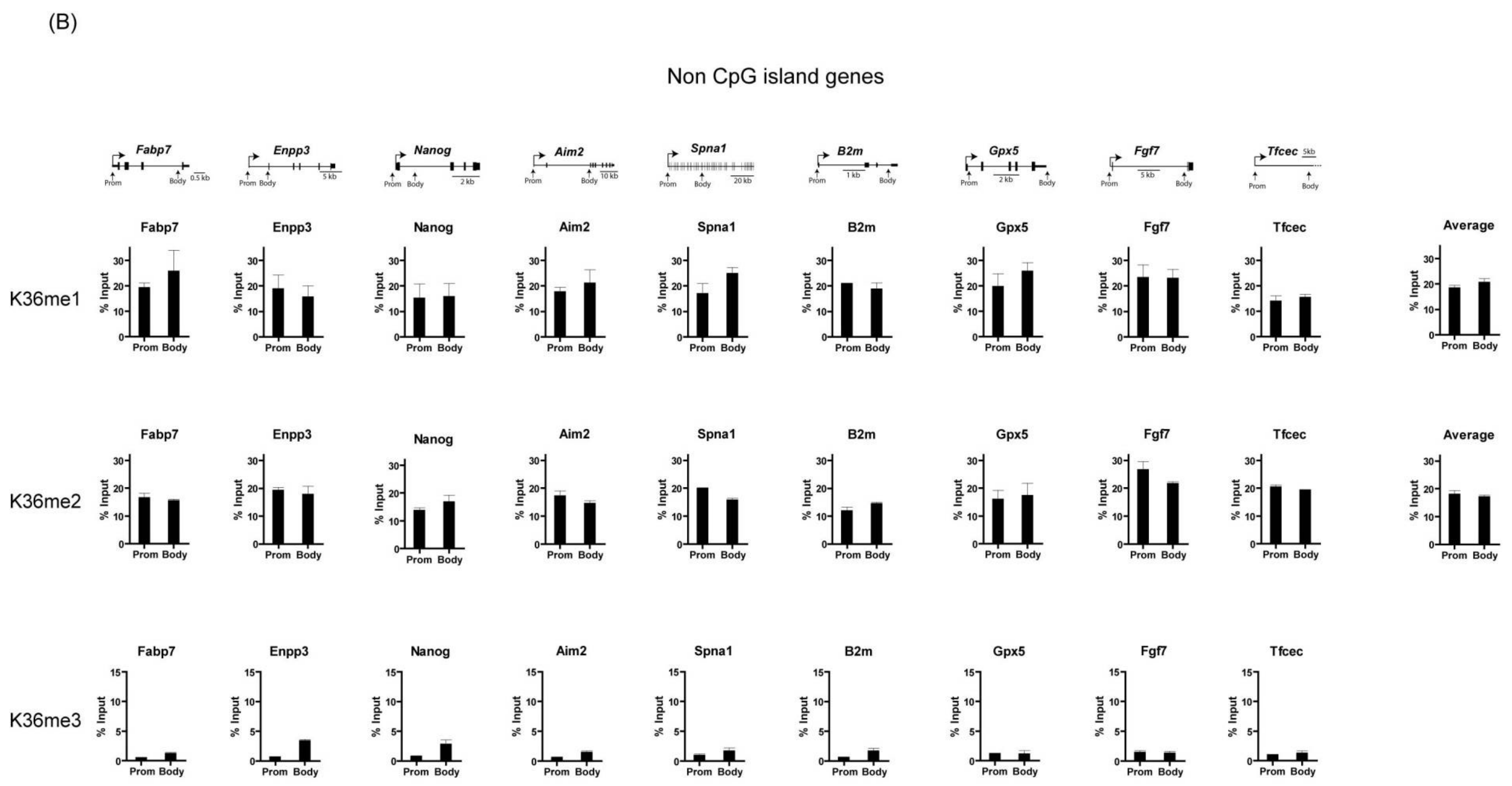

Figure S5 (continued)

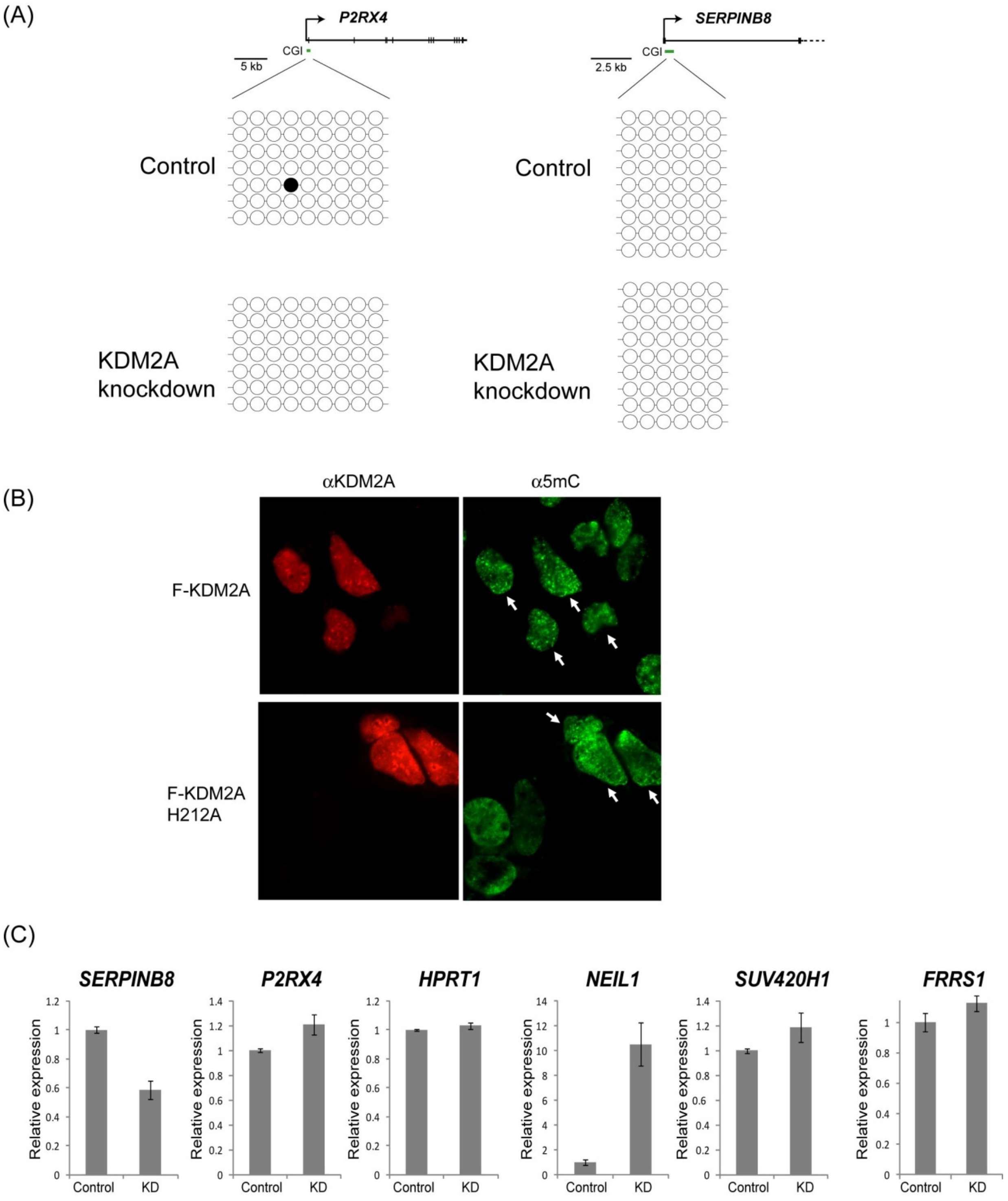
(A)
P2RX4
CGI
5 kb
Control
KDM2A knockdown
SERPINB8
CGI
2.5 kb
Control
KDM2A knockdown
(B)
αKDM2A
α5mC
F-KDM2A
F-KDM2A H212A
(C)
SERPINB8
P2RX4
HPRT1
NEIL1
SUV420H1
FRRS1
Relative expression
Control
KD

Figure S6

Supplemental

Figure S1

(A) Non linear fitting of the SPR signal at equilibrium with the Langmuir binding isotherm illustrating the interaction of KDM2A with a 75bp oligonucleotide probe containing 1 CpG. The point of intercept between the curve and the vertical line represents the K_D.

(B) The same analysis in (A) was applied to data from a probe containing 6 CpG's.

Figure S2

(A) KDM2A immunoprecipitation from V6.5 mouse ES cell nuclear extract followed by western blot analysis. Full length KDM2A protein migrates by SDS-page at an expected size of 132 kDa. Smaller immunoreactive protein species (marked with *) represent KDM2A degradation products. The antibody heavy chain is marked with **.

(B) Western blot of whole cell extract from control cells (left hand lane) and cells expressing exogenous Flag-tagged full length KDM2A (right hand lane). The Flag specific antibody detects smaller immunoreactive species (marked with *) of the same size as those observed in (A) indicating that these are genuine KDM2A degradation products.

(C) KDM2A was immunoprecipitated from V6.5 mouse ES cell formaldehyde crosslinked chromatin under ChIP wash conditions followed by western blot analysis indicating specific immunoprecipitation of the full length protein. The KDM2A band is only visible in the enriched IP sample as KDM2A is not detectable in dilute whole cell ChIP extracts. The antibody heavy chain is marked with **.

(D) Indirect immunofluorescence with anti-KDM2A antibodies (red) and DAPI staining of DNA (blue). Endogenous KDM2A staining is found throughout the nucleoplasm in *Dnmt1* +/+ MEFs (upper panels). DAPI bright staining punctate foci mark regions of densely methylated pericentromeric heterochromatin (lower panels).

(E) In *Dnmt1-/-* MEFs that are nearly devoid of CpG methylation, KDM2A localizes to the hypomethylated pericentromeric DAPI bright staining repeat regions (compare upper and lower panels). In *Dnmt-/-* MEFs a proportion of cells lack clear DAPI foci (indicated with asterisk) and in these instances focal KDM2A staining is not observed. The subpopulation of cells that lack clear DAPI bright staining foci is specific to the *Dntmt1*-/- MEFs and may result from structural alterations caused by prolonged culture in the absence of DNA methylation. In both (D) and (E) the co-localization of KDM2A foci with DAPI bright staining foci was counted in an additional 180 (D) and 172 (E) nuclei and is indicated as a percentage of total cells counted.

(F) Indirect immunofluorescence with anti-Flag antibodies illustrating localization of exogenous KDM2A (green, top panel) and DNA via DAPI staining (blue, bottom panel) in *Dnmt* -/- MEFs. In transiently transfected cells, exogenous KDM2A localizes to DAPI bright foci. The co-localization of KDM2A foci with DAPI bright foci was counted for 213 transfected nuclei and is indicated as a percentage.

(G) KDM2A with catalytically inactive JmjC domain still localizes to DAPI bright foci.

(H) KDM2A-ΔCxxC does not localize to DAPI bright foci.

(I) KDM2A-ΔPHD localizes to DAPI bright foci.

(J) Indirect immunofluorescence with a 5mC-specific antibody comparing the *Dnmt1* +/+ and -/- cell lines verifying the absence of DNA methylation in *Dnmt -/-* MEFs (right panel). *Dnmt +/+* MEFs show strong abundant focal nuclear staining (left panel, 97% of nuclei, n=190) and *Dnmt -/-* MEFs exhibit very little or no focal nuclear staining (right panel, 3% of nuclei, n=177). Dotted white lines have been drawn around the nuclei in each image.

Figure S3

Using chromatin from mouse ES cells, ChIP was performed with a KDM2A-specific antibody and two groups of genes were analyzed by quantitative real time PCR. Genes with CpG island

promoters are shown in the top panel and genes with non-CpG island promoters in the bottom panel. A schematic for each gene is shown above the ChIP data. Vertical black bars represent exons. CpG islands are indicated by green horizontal bars. For each gene, a promoter (prom) and body primer set was designed, as indicated on the schematic. Enrichment obtained with an antibody against KDM2A (black) and HA (white) is shown for each primer set. CpG island promoters are enriched for KDM2A when compared to gene body regions. Non-CpG island promoters show no KDM2A enrichment when compared to body regions. ChIP experiments represent at least biological duplicates with error bars indicating SEM.

Figure S4

(A) to (D) KDM2A ChIP-seq analysis for a selection of genomic regions in mouse ES cells (red, top panel of each figure) with comparative analysis from input material (blue, lower panel of each figure). Annotated genes in each region are illustrated above the sequence traces with arrows indicating direction of genes, vertical black bars corresponding to exons, and CpG islands (CGIs) represented by green bars.

(E) Average KDM2A tag density at expressed and silent CpG island promoters compared to expressed and silent non-CpG island promoters. The X-axis shows position relative to transcription start site (TSS) of each category of gene. Importantly, KDM2A tag density at both silent and expressed CpG island genes is enriched in comparison to silent or expressed non-CpG island genes, suggesting that KDM2A binding is determined by non-methylated CpG content at these genes rather than the underlying transcriptional status of the gene. The enrichment of KMD2A at some expressed non-CpG island genes is likely due to inclusion of some non-methylated island genes that fall below the threshold for inclusion in the algorithm defined CpG island set.

(F) Average KDM2A tag density at GC-rich compared to GC-poor promoters. As an alternative to using the CpG island algorithm to define promoter type, gene promoters were categorized as either GC-rich or GC-poor as increased GC content is a feature of experimentally identified non-methylated islands. GC-rich promoters show clear enrichment of KDM2A tag density compared to GC-poor promoters.

(G) As observed in (E) KDM2A binding occurs at GC rich promoters whether silent or expressed, supporting the observation that KDM2A nucleation occurs independently of the transcriptional state of the associated gene. The clear separation of KDM2A bound promoters based on GC content also supports the contention that KDM2A binding seen at some expressed non-CpG islands genes in (E) is likely due to inclusion of non-methylated island regions due to algorithm based limitations.

(H) KDM2A tag density at algorithm defined CpG island and non-CpG island clusters. The magnitude of KDM2A tag density is greater at algorithm defined CpG island clusters than those that fall outside of the cutoffs for inclusion in the bioinformatically defined CpG island set.

(I) Average KDM2A tag density at genes with strong CpG island, weak CpG island, and non-CpG island promoters. Again, KDM2A density correlates with the strength of the underlying CpG island.

(J) A graph showing GC content plotted against the fraction of total KDM2A clusters. The vertical red line indicates average GC content in mouse genome.

(K) A graph showing CpG observed/expected ratio plotted against the fraction of total number of KDM2A clusters (N=40,227). The vertical red line indicates average CpG observed/expected ratio in the mouse genome (Simmen, 2008). In both (J) and (K), KDM2A clusters are colored according to whether they correspond to CpG island or non-CpG island regions as defined bioinformatically using the UCSC genome browser CpG island track.

(L) to (Q) Bisulfite sequencing at a panel of KDM2A clusters not associated with algorithm-defined CpG islands. Empty and filled circles represent non-methylated and methylated CpG dinucleotides, respectively.

Figure S5

(A) ChIP was performed in mouse ES cells using antibodies specific for H3K36me1 (top panel), H3K36me2 (middle panel) and H3K36me3 (bottom panel). qPCR was carried out using promoter and body primer sets for 9 different CpG island genes, as depicted above the ChIP data. Average H3K36me1 and H3K36me2 values are shown in the extreme right hand panel. When compared to gene body regions, CpG island promoters show significant depletion of H3K36me1 (*$p<0.05$), and in particular H3K36me2 (**$p<0.01$). Genes at which levels of H3K36me2 are low in the promoter and body regions (for example *Rae1* and *Brd2*) show high levels of H3K36me3 in the body region. In these instances, depletion of H3K36me2 in the body possibly reflects conversion of H3K36me2 to the me3 modification state. ChIP data is normalized for his H3 occupancy and is derived from biological duplicate experiments. Error bars represent SEM.

(B) A panel of 9 non CpG island genes was analyzed as for (A). At non CpG island genes, H3K36me1 and H3K36me2 show no significant difference between the promoter and body regions ($p>0.05$).

Figure S6

(A) Bisulfite sequencing was used to investigate CpG methylation at the CpG islands of *P2RX4* and *SERPINB8* in control cell line (top panel) and KDM2A knockdown cell line (bottom panel). Empty and filled circles represent non-methylated and methylated CpG dinucleotides, respectively. Both CpG islands remain non-methylated in KDM2A knockdown cell line.

(B) Indirect immunofluoresence with anti-KDM2A and anti-5mC antibodies in cells transiently over-expressing KDM2A (top panels) or KDM2A with a point mutation in the JmjC domain (H212A) that abrogates histone lysine demethylase activity (bottom panels). Over-expression of KDM2A does not affect global 5mC levels.

(C) Gene expression analysis by quantitative RT-PCR for a panel of 6 genes that were previously subjected to ChIP analysis in Figure 5. In all instances, expression is normalized to RPS27A, a housekeeping gene with a non CpG island promoter. For each gene, expression in the control cell line is set to 1, with expression in the KDM2A knockdown (KD) cell line shown relative to this. Data is from biological duplicate experiments. Error bars represent SEM.

Table S1. KDM2A ChIP-seq statistics

Total number of KDM2A clusters	40,227
Percentage of KDM2A clusters that overlap CpG islands	34.1%
Percentage of CpG islands that overlap KDM2A clusters	92.0%
Percentage of transcription start sites within KDM2A clusters	49.6%
Percentage of CpG island transcription start sites* within KDM2A clusters	95.9%

* Transcription start sites are considered to be 'CpG island transcription start sites' if they are encompassed by a CpG island.

Supplemental Experimental Procedures

DNA constructs

The full length human KDM2A was PCR amplified from cDNA clone KIA1004 (a gift from T. Nagase) and inserted via ligation independent cloning (LIC) into a pLion2 eukaryotic expression vector (Addgene Plasmid 1730) that has been modified to express an N-terminal flag tag and contain LIC hybridization sites. Mutations in the JmjC domain (H212A and D214A) and ZF-CxxC domain (K601A) were introduced into the wild type KDM2A construct using the Quikchange mutagenesis XL kit (Stratagene). The ΔZF-CxxC and ΔPHD cDNAs (a gift from Y. Zhang) were PCR amplified and cloned into Flag tagged LIC adapted pLion2 vector. The prokaryotic expression vector used to produce his-tagged KDMA-ZF-CxxC domain (AA 544-648) was generated by PCR amplification from the KIAA1004 cDNA and cloned into the *BglII/NotI* site of pet30b (Novagen). The prokaryotic expression vector used to produce His-KDM2A-StrepII (AA 1-747) and mutant derivatives were PCR amplified from the mammalian expression vectors and cloned into his-tagged LIC adapted pNIC28 vector (a gift from U. Oppermann) with a StrepII tag being introduced into C-terminus of the construct during PCR amplification. All PCR generated constructs were verified by sequencing. The KDM2A knockdown construct was generated by cloning a target specific short hairpin DNA sequence (atcactggagttcctatagtac) into the pLMP miR30 based knockdown vector (a gift from R. Dickins and S. Lowe).

Cell culture

Murine V6.5 ES cells (C57BL/6 (F) x 129/sv (M)) were cultured on inactivated MEFs in DMEM supplemented with 15% FCS, leukemia-inhibiting factor, penicillin/streptomycin, L-glutamine and non-essential amino-acids. Prior to use in ChIP experiments, V6.5 cells were cultured for two passages under feeder-free conditions on 0.2% gelatin (as described in(Bernstein et al.,

2006)). Mouse adult lung fibroblasts, *Dnmt1*+/+ MEFs, and *Dnmt1*-/- MEFs (a gift from H. Cedar) were cultured in DMEM supplemented with 10% FCS and penicillin/streptomycin. Hela cells were grown in DMEM supplemented with 10% FCS and penicillin/streptomycin. After transfection of the pLMP short hairpin silencing vector into HeLa cells, stable KDM2A knockdown and control cell lines were selected in 1µg/ml puromycin and recovered as clonal isolates. U2OS were cultured in DMEM supplemented with 10% FCS and penicillin/streptomycin and transfected with Fugene HD (Roche).

Chromatin immunoprecipitation

For KDM2A ChIP, cells were fixed for 1 hour in 2 mM EGS, followed by 15 minutes in 1% formaldehyde, while for histone modification ChIP cells were fixed for 10 minutes in 1% formaldehyde alone. In both cases, formaldehyde was quenched by the addition of glycine to a final concentration of 125 μM. Cells were lysed in 1% SDS, 10 mM EDTA, 50 mM Tris-HCl (pH 8.1) plus complete protease inhibitors, and sonication was performed using a BioRuptor sonicator (Diagenode) to produce fragments of approximately 500 bp. Immunoprecipitation was performed overnight at 4°C with approximately 3 µg of antibody and chromatin corresponding to 5 x 10^6 cells (KDM2A ChIP) or 1 x 10^5 cells (histone modification ChIP), in 1% triton-x-100, 1 mM EDTA, 20 mM Tris-HCl (pH 8) and 150 mM NaCl. Commercial antibodies used for ChIP were anti-Histone H3K36me1 (Abcam 9048), anti-Histone H3K36me3 (Abcam 9050) and anti-Histone H3 (Abcam 1791) and the H3K36me2 antibody was generated by immunizing rabbits with a synthetic peptide (apatggv (Kme2)kphryrpg) corresponding to H3K36me2, and the antibody was purified on peptide affinity columns. Antibody bound proteins were isolated on protein A agarose beads (Repligen), washed extensively, eluted, and cross-links reversed according to the Upstate protocol. Samples were then sequentially treated with RNase and proteinase K before being purified on a PureLink PCR micro column (Invitrogen). Real-time qPCR was performed using Sybr Green (Quantace) on a Rotor-Gene 6000 (Corbett). For all

histone modification ChIP experiments, enrichment values for specific modifications were normalized to enrichment values for histone H3. Where indicated statistical analysis of ChIP data was performed by unpaired t-tests. Primer sets used for qPCR are available upon request.

ChIP DNA preparation for Solexa Sequencing

ChIP DNA was prepared for Solexa 2G sequencing by blunting the DNA with a mixture of T4 DNA polymerase, Klenow DNA polymerase, and T4 PNK (NEB) according to manufacturer's instruction. dA overhangs were then added and Illumina adapters ligated. Adapter-ligated DNA was subject to 18 cycles of PCR before size selection by agarose gel electrophoresis. Amplified DNA was purified using the Qiaquick gel extraction kit (Qiagen). The purified DNA was quantified both with an Agilent Bioanalyzer and Invitrogen Qubit and diluted to a working concentration of 10 nM prior sequencing. Sequencing on a Solexa 2G instrument was carried out according to the manufacturer's instructions.

Bisulfite Sequencing

Bisulfite conversion of DNA was performed using the EZ DNA Methylation-Gold Kit (Zymo Research). PCR-amplified DNA was cloned into pGEM-T Easy (Promega) and sequenced. Sequenced clones were analysed using the web-based tool QUMA (http://quma.cdb.riken.jp/) (Kumaki et al., 2008).

Immunofluoresence

Cells were fixed for 20 min in 4% paraformaldehyde (24 hours after transfection (Fugene HD) where KDM2A was expressed exogenously), washed 3 times with PBS, and subsequently permeabilized for 20 min in 0.5% TritonX-100/PBS. Permeabilized cells were washed 2 times in PBS and blocked in 3% BSA/PBS for 30 min. Cells were incubated with primary antibody in a humidified chamber for 1-3 hours using the Flag mouse monoclonal M2 antibody (Sigma) at a

dilution of 1:1000, the KDM2A rabbit polyclonal antibody at a dilution of 1:100, the H3K36me2 rabbit polyclonal antibody at 1:100, and the mouse monoclonal antibody against 5mC (Eurogentec) at 1:100. After primary antibody incubation, cells were washed 3 times with PBS and incubated with FITC or Rhodamine conjugated secondary antibodies (Jackson ImmunoResearch Laboratories). Cells were washed twice with PBS, stained with 4,6-diamidino-2-phenylindole dihydrochloride (DAPI) and mounted on glass slides in fluorescent mounting medium (DAKO). Slides were analyzed on an AxioSkop fluorescent microscope (Zeiss).

Protein Expression and Purification

Expression and purification of KDM2A ZF-CxxC domain and KDM2A 1-747 constructs were performed as previously described (Klose and Bird, 2004) except for the KDM2A 1-747 constructs where the elution from the Ni-NTA was directly loaded onto a pre-washed StrepTactin Superflow column (IBA). The sample was allowed to flow over the column several times and the resin was then washed with wash buffer (100 mM Tris-Cl pH 8, 150 mM NaCl) and eluted in elution buffer (100 mM Tris-Cl pH 8, 150 mM NaCl, 2.5 mM desthiobiotin). Purified protein was dialyzed overnight in BC100 buffer (50 mM Hepes pH 7.9, 100 mM KCl, 10% glycerol, 0.5 mM DTT) and stored at -80°C.

EMSA and SPR probes

A short randomly generated DNA probe (GTAGGCGGTGCTACACGGTTCCTGAAGTG) containing two CpGs was assembled by annealing complementary oligo nucleotides and then end labeled with ATP [γ-^{32}P] (Perkin Elmer) and T4 polynucleotide kinase (Fermentas). The probes were then purified on a nucleotide removal kit (Qiagen) and used for experiments in Figure 1B. A longer CpG DNA containing probe was generated by PCR amplification of a 147 base pair fragment from the pGEM-3Z 601 plasmid (a gift from T. Owen-Hughes and J. Widom)

for experiments in Figure1D (sequence available on request). Amplified DNA was then purified on a Resource Q anion exchange column (GE Healthcare). A small aliquot of purified DNA was methylated overnight with *SssI* DNA methyltransferase (NEB) and purified using the QIAquick PCR purification kit (QIAGEN). A small fraction of each non-methylated and methylated DNA samples was radioactively end-labeled using T4 polynucleotide kinase (Fermentas) and ATP [γ-^{32}P] (Perkin Elmer). The labeled DNA was then purified using the QIAquick PCR purification kit (QIAGEN) and stored at -20°C. For SPR experiments three 75 base pair oligonucleotides probes containing 0, 1 or 6 evenly spaced CpG sites were generated with a 5' biotin moiety. The same random DNA sequence was used as a basis for each probe and the indicated CpG density was substituted into this sequence (sequence available on request).

Electrophoretic mobility shift assay (EMSA)

EMSA reactions were assembled in binding buffer (4% Ficoll 400, 20 mM Hepes 7.9, 150 mM KCl, 1 mM EDTA, 0.5 mM DTT, 25 ng/µl poly-dAdT competitor DNA). The protein sample was incubated in EMSA buffer for 10 min at room temperature prior to addition of the radiolabelled probe. The mixture was allowed to incubate for 20 min at room temperature and loaded onto a 0.8% agarose gel in 0.5X TBE. The gel was run at 100V, 4°C, subsequently dried onto a DE81 anion exchanger paper (Whatman) via a vacuum driven gel-dryer (Amersham) and exposed to a phosphorimager screen overnight. The screen was then scanned using a fluorescent image analyser (FLA-7000, Fujifilm).

Surface plasmon resonance

Surface plasmon resonance measurements were determined using a Biacore T100 machine (GE Healthcare). Three different oligonucleotide probes containing respectively 0, 1 or 6 CpG sites were used. 2.5 pmol of each probe diluted in coupling buffer (10mM Hepes pH 7.9, 150 mM NaCl, 3 mM EDTA, 0.005% Tween 20) was immobilized on the surface of a streptavidin

(SA) coated sensor chip (GE Healthcare) at a flow rate of 5μl/min. All flow cells were then washed with 200μM biotin in 1X binding buffer (10 mM Hepes pH 7.9, 150 mM NaCl, 1 mM DTT) to block all unbound streptavidin sites. Serial dilutions (3 μM, 1.5 μM, 750 nM, 375 nM, 187.5 nM, 0 nM) of KDM2A 1-747 protein were prepared in 1X binding buffer containing 100 ng/μl of poly (dAdT) and 20 μl of each dilution was injected at a flow rate of 5 μl/min. Bound proteins were allowed to dissociate in binding buffer for 10 min followed by an elution step with 20 μl of regeneration buffer (2.5 M NaCl, pH 5.3). The K_D value was determined for the 1 and 6 CpG probes using a Langmuir binding curve.

Sequencing data analysis

Sequenced tags of 51 bp in length were mapped to the mouse genome (mm9) using Bowtie aligner (Langmead et al., 2009). Only uniquely mapped tags with no more than two mismatches were retained. Positions in genome with the numbers of mapped tags above the significance threshold defined by a *Z*-score of 7 were identified as anomalous, potentially resulting from amplification bias, and the tags mapped to those positions were discarded. The final sets used for further analysis comprised 10,042,076 KDM2A ChIP tags and 9,760,426 input tags. The characteristic sizes of DNA fragments in the ChIP and input samples were estimated to be 100 bp and 90 bp respectively using the strand cross-correlation analysis(Kharchenko et al., 2008). Since the positions of sequenced tags correspond to 5'-ends of the DNA fragments, these positions were shifted by the half of the characteristic fragment size towards the fragment 3'-ends to represent centers of the DNA fragments. The positions from positive and negative DNA strands were combined. To determine regions of significant tag enrichment of the ChIP sample over the input, we calculated the tag fold-enrichment in overlapping 500-bp windows. The continuous regions of enrichment were determined based on Z-score threshold of 3. Enriched regions shorter than 250 bp were discarded, and the clusters separated by less than 500 bp were merged. One percent of the genes with the highest and lowest tag counts were not taken

for averaging. The profiles were smoothed in a 100 bp running window. The annotation from the UCSC Genome Browser was used to determine the boundaries of CpG islands (Karolchik et al., 2003). CpG island promoters were determined as promoters of the genes that have their TSS encompassed by CpG islands. All other genes were assigned to the non-CpG island group. To determine the sets of GC-rich and GC-poor sequences at TSS, the thresholds of 0.55 and 0.40 were used to stratify 4-kb fragments centered at TSS by the GC-content. To avoid possible distortions, genes shorter than 1 kb and overlapping genes were filtered out for the calculations of the average tag density profiles around TSS. The groups of the 'strong' and 'weak' CpG islands were defined based on the GC-content and CpG observed-to-expected ratio in the island. The selected threshold values correspond to the means for all the CpG islands in the genome and were equal to 0.65 for the GC-content and 0.9 for the CpG o/e ratio. The islands that had both GC-content and CpG o/e ratio above (below) the thresholds were identified as strong (weak). There were 3,506 strong islands, 3,547 weak islands, and 8,972 islands were not included in any group.

Microarray and Data analysis

Total RNA was extracted from the KDM2A knockdown and control cell lines in biological replicates. The RNA labeling and hybridization to Agilent 4 × 44 K human gene expression microarrays (Agilent Technologies, Inc., Santa Clara, CA) was carried out by Oxford Gene Technology (Oxford, UK). Hybridizations were performed for two biological replicates for each sample. Microarray data were background-corrected and quantile-normalized between the arrays using Bioconductor packages Agi4x44PreProcess and limma (Smyth, 2004). The individual probe data were summarized for all the unique transcripts present on the array (20,240 transcripts in total). Fold-change and statistical significance were estimated for the mean expression values for the replicate sets.

Supplemental References

Karolchik, D., Baertsch, R., Diekhans, M., Furey, T. S., Hinrichs, A., Lu, Y. T., Roskin, K. M., Schwartz, M., Sugnet, C. W., Thomas, D. J., *et al.* (2003). The UCSC Genome Browser Database. Nucleic Acids Res *31*, 51-54.

Kharchenko, P. V., Tolstorukov, M. Y., and Park, P. J. (2008). Design and analysis of ChIP-seq experiments for DNA-binding proteins. Nat Biotechnol *26*, 1351-1359.

Klose, R. J., and Bird, A. P. (2004). MeCP2 behaves as an elongated monomer that does not stably associate with the Sin3a chromatin remodeling complex. J Biol Chem *279*, 46490-46496.

Kumaki, Y., Oda, M., and Okano, M. (2008). QUMA: quantification tool for methylation analysis. Nucleic Acids Res *36*, W170-175.

Langmead, B., Trapnell, C., Pop, M., and Salzberg, S. L. (2009). Ultrafast and memory-efficient alignment of short DNA sequences to the human genome. Genome Biol *10*, R25.

Simmen, M. W. (2008). Genome-scale relationships between cytosine methylation and dinucleotide abundances in animals. Genomics *92*, 33-40.

Smyth, G. K. (2004). Linear models and empirical bayes methods for assessing differential expression in microarray experiments. Stat Appl Genet Mol Biol *3*, Article3.
